# Neural Cadherin Plays Distinct Roles for Neuronal Survival and Axon Growth under Different Regenerative Conditions

**DOI:** 10.1523/ENEURO.0325-20.2020

**Published:** 2020-11-13

**Authors:** Márcio Ribeiro, Konstantin Levay, Benito Yon, Ana C. Ayupe, Yadira Salgueiro, Kevin K. Park

**Affiliations:** Miami Project to Cure Paralysis, Department of Neurosurgery, University of Miami Miller School of Medicine, Miami, FL 33136

**Keywords:** axon growth, axon regeneration, cadherins, NCAD, PTEN, retinal ganglion cells

## Abstract

Growing axons in the CNS often migrate along specific pathways to reach their targets. During embryonic development, this migration is guided by different types of cell adhesion molecules (CAMs) present on the surface of glial cells or other neurons, including the neural cadherin (NCAD). Axons in the adult CNS can be stimulated to regenerate, and travel long distances. Crucially, however, while a few axons are guided effectively through the injured nerve under certain conditions, most axons never migrate properly. The molecular underpinnings of the variable growth, and the glial CAMs that are responsible for CNS axon regeneration remain unclear. Here we used optic nerve crush to demonstrate that NCAD plays multifaceted functions in facilitating CNS axon regeneration. Astrocyte-specific deletion of NCAD dramatically decreases regeneration induced by phosphatase and tensin homolog (PTEN) ablation in retinal ganglion cells (RGCs). Consistent with NCAD’s tendency to act as homodimers, deletion of NCAD in RGCs also reduces regeneration. Deletion of NCAD in astrocytes neither alters RGCs’ mammalian target of rapamycin complex 1 (mTORC1) activity nor lesion size, two factors known to affect regeneration. Unexpectedly, however, we find that NCAD deletion in RGCs reduces PTEN-deletion-induced RGC survival. We further show that NCAD deletion, in either astrocytes or RGCs, has negligible effects on the regeneration induced by ciliary neurotrophic factor (CNTF), suggesting that other CAMs are critical under this regenerative condition. Consistent with this notion, CNTF induces expression various integrins known to mediate cell adhesion. Together, our study reveals multilayered functions of NCAD and a molecular basis of variability in guided axon growth.

## Significance Statement

Growing axons often travel long distances and migrate along explicit pathways to reach their targets. Cell adhesion molecules (CAMs), including cadherins, play vital roles in these processes during development. However, it remains unclear whether the same factors are involved for the adult axons after injury. This study used knock-out (KO) mice to demonstrate that ablation of NCAD in astrocytes or retinal ganglion cells (RGCs), prevents regeneration and cell survival induced by phosphatase and tensin homolog (PTEN) deletion. In contrary, NCAD deletion has negligible effects on the regeneration and survival induced by cytokines, suggesting that distinct CAMs control axon adhesion and growth under different regenerative conditions. Together, our study illustrates cadherins’ versatile functions in the injured CNS, and points to distinct mechanisms that shape directed axon growth.

## Introduction

The projection neurons in the adult CNS normally do not regenerate their axons after an injury. However, these neurons can regenerate, if they are given the appropriate stimulation (Chierzi and Fawcett, 2001). For example, administering combinations of growth factors, including insulin growth factor 1 (IGF1; Duan et al., 2015; Bei et al., 2016), interleukin-6 (IL-6; Fischer, 2017), ciliary neurotrophic factor (CNTF; Park et al., 2004; Leaver et al., 2006), and osteopontin (Bei et al., 2016; Anderson et al., 2018), stimulates regeneration of CNS axons. Other studies have shown that modulating distinct genes within the neurons, including signal transducers and activators of transcription factor 3 (*Stat3*; Moore and Goldberg, 2011; Luo et al., 2016), histone deacetylase 5 (*Hdac5*; Cho and Cavalli, 2012), *Cacna2d2* (Tedeschi et al., 2016), *Lin28a* (Wang et al., 2018; Nathan et al., 2020), phosphatase and tensin homolog (*Pten*; Park et al., 2008), or Kruppel-like factors (*Klfs*; Moore et al., 2009), can enhance neurons’ intrinsic axon growth ability. Numerous studies have demonstrated that environmental factors also contribute to axon regeneration. Among such factors, astrocytes have been studied extensively (Silver et al., 2014; Anderson et al., 2016); astrocytes are not only a source of growth factors, but they also serve as physical scaffolds and provide pathways for CNS axons to adhere and migrate (Tom et al., 2004; Rigby et al., 2020). For example, in the spinal cord of PTEN-deleted mice, regenerating axons almost always grow along the astrocytic processes that span the lesion site (i.e., astrocytic “bridges”; Liu et al., 2010; Zukor et al., 2013).

Growing axons in the CNS must often travel long distances and migrate along specific pathways to reach their targets. During development, these axons are guided by various cell adhesion molecules (CAMs; Walsh and Doherty, 1997; Blackmore and Letourneau, 2006; Kamiguchi, 2007), expressed on the surface of glial cells or other neurons. In the mature CNS, however, regenerating axons often travel circuitously and never reach their destinations, indicating that the cellular and molecular factors that once operated during development do not necessarily remain present or may be inadequate for the adult axons. CAMs are a large group of proteins located on the cell surface, involved in the binding of cells to glial cells or extracellular matrix (ECM) proteins (Kamiguchi, 2007; Hillen et al., 2018). CAMs are categorized broadly into four superfamilies: the immunoglobulin superfamily of CAMs (IgCAMs), cadherins, integrins, and the superfamily of C-type lectin-like domain proteins (CTLDs; Chothia and Jones, 1997; Graham and Duan, 2020). Within the cadherins, neural cadherin [NCAD; also known as cadherin-2 (CDH2)] is expressed in neuronal cells as well as in glial cells of the CNS in vertebrate embryos (Radice et al., 1997). Several studies have demonstrated that NCAD plays a critical role in guiding the migration of neurites on astrocytes during development (Hansen et al., 2008). Transfection with NCAD promotes the outgrowth of chicken embryonic optic axons on monolayer cultures (Matsunaga et al., 1988). An *in vitro* study has shown that the genetic deletion of NCAD in cultured astrocytes impairs the formation and extension of sensory neurons’ neurites on astrocytes (Ferguson and Scherer, 2012). Other studies have shown that cadherins play a critical role in promoting axon fasciculation, allowing axons with similar cadherins on their surfaces to cluster together, and grow toward their common targets (Bastiani et al., 1987; Missaire and Hindges, 2015; Bruce et al., 2017). However, while a great amount of knowledge has been gathered about cadherins’ contributions to axon growth and guidance during development, it is unclear whether the same factors control this process during axon regeneration in adulthood. In this study, we used optic nerve crush and gene knock-out (KO) strategies in adult mice to investigate the extent to which astrocytes facilitate axon adhesion and regeneration, and whether NCAD and other CAMs mediate axon regeneration under two different conditions, PTEN deletion and CNTF overexpression.

## Materials and Methods

### Animals

All animal experimental procedures were performed in compliance with protocols approved by the Institutional Animal Care and Use Committee (IACUC) at the University of Miami. Animals used were C57BL/6J (The Jackson Laboratory, stock number 000664), NCAD^f/f^ (The Jackson Laboratory, stock number 007611), GFAP-CreERT (a gift from Ken D. McCarthy, University of North Carolina; Casper et al., 2007), PTEN^f/f^ (The Jackson Laboratory, stock number 006440), Rosa26 loxP-STOP-loxP-tdTomato (R26-tdTomato, a gift from Fan Wang, Duke University, Durham), and Rosa26-STOP-EYFP (The Jackson Laboratory, stock number 006148). All animals were housed in a viral antigen-free facility and kept under standard 12/12 h light/dark conditions. For all surgical procedures, mice were anaesthetized with ketamine and xylazine. For analgesia, buprenorphine (0.05 mg/kg) was administered postoperatively. Tamoxifen injections. Tamoxifen stock solutions (50 mg/ml) were prepared from 1 g of Tamoxifen Free Base MP Biomedicals (fisher scientific) dissolved in a sterile mixture of 10% ethanol plus 90% sunflower oil. The tamoxifen solution was placed at 55°C in a water bath during 3 h while protected from light and vortexed every hour and then stored at −20°C until use. Young adult mice (five to six weeks old) received daily intraperitoneal injections of tamoxifen (0.124 mg/g body weight) for five consecutive days.

### Cloning and generation of adeno-associated viruses (AAVs)

To suppress PTEN expression, we adopted a shRNA strategy, based on SIBR vectors in which shRNA is located in an intron and flanked by sequences derived from mir155, an endogenous intronic shRNA (Chung et al., 2006; Yungher et al., 2015). To maximize the probability of effectively targeting PTEN, four separate shRNA sequences, each targeting a different region of PTEN were concatenated in a single plasmid, which was then used to produce AAV (AAV-shPTEN). Four sequences that target both mouse and rat PTEN were designed using siDIRECT website and design rules: four targeted sequences for PTEN are: GCAGAAACAAAAGGAGATATCA;GATGATGTTTGAAACTATTCCA;GTAGAGTTCTTC CACAAACAGA;GATGAAGATCAGCATTCACAAA. Oligonucleotides encoding hairpin loops that included these sequences and deliberate mis-matches in the non-target strand were synthesized, annealed, inserted into the SIBR knock-down vector, and concatenated into a single plasmid as described. A region of the SIBR knock-down vector comprising the ubiquitin promoter, intronic sequences, knock-down cassette, and EGFP open reading frame was cloned into an AAV-compatible plasmid (AAV-MCS, Stratagene), from which the CMV promoter, intron and MCS were removed. pAAV-RC (Stratagene) that encodes the AAV2 genes (rep and cap) and the helper plasmid (Stratagene) that encodes E2A, E4, and VA were used for co-transfection in 293T cells to generate recombinant AAV. Plasmids were then used to produce AAV2 (1–4 × 1013 particles/ml). To construct AAV expressing a secretable form of CNTF, an AAV-compatible SIBR vector was created by PCR-amplifying the knock-down cassette of a SIBR vector with primers that created 5′ Mlu1 (ACGCGTTTAAACTGGCCTCCGCGCC) and 3′ ClaI (ccgccgATCGATTCACTTGTACAGCTCGTCCA) sites (Yungher et al., 2015). This cassette was inserted into a Stratagene AAV plasmid, replacing the CMV promoter and B-globin intron. The resulting AAV-SIBR plasmid was then modified via bridge PCR to create KpnI and BglII sites to flank the EGFP open reading frame. Plasmid DNA encoding human CNTF was purchased from OpenBiosystems (accession BC068030) and the open reading frame was amplified using a forward primer that incorporated both a 5′ KpnI restriction site and the NGF signal peptide sequence (GGTACCATGTCCATGTTGTTCTACACTCTGATCACAGCTTTTCTGATCGGCATACAG GCGGCTTTCACAGAGCATTCACCGC) and a reverse primer that incorporated 3′ BglII site (AGATCTCTACATTTTCTTGTTGTTAGCAA). PCR-amplified CNTF was then used to replace the EGFP ORF in the AAV-SIBR vector via standard restriction digest and ligation. All enzymes were purchased from New England Biolabs. Plasmids were then used to produce AAV serotype2 (1–4 × 1013 particles/ml). For making AAV2 expressing Cre recombinase, the cDNA of Cre was inserted downstream of the CMV promoter/β-globin intron enhancer in the plasmid pAAV-MCS (Stratagene), containing the AAV2 inverted terminal repeats and a human growth hormone poly A signal. Plasmids were then used to produce AAV2 (1–4 × 1013 particles/ml).

### Intravitreal injection

For AAV injection, ∼2-μl volume was injected using a Hamilton syringe (Hamilton 80900) coupled with a fine glass micropipette inserted into the posterior chamber of the eye and deliberately angled to avoid damage to the lens. To label regenerating axons anterogradely, we injected 2 μl of Alexa Fluor 488-conjugated or Alexa Fluor 555-conjugated cholera toxin β subunit (CTB; 2 μg/μl; ThermoFisher C22841 and C22843) 3 d before killing. AAV-CRE and AAV-shPTEN were injected two weeks before optic nerve crush to allow sufficient time for the buildup of the transgenes and subsequent gene KO. AAV-CNTF injection was done one week after the last tamoxifen injection, and optic nerve crush done 3 d after AAV-CNTF injection.

### Optic nerve crush

The optic nerve was crushed unilaterally and intraorbitally using a pair of forceps (#5 Dumont, Fine Science Tools) for 10 s, ∼1 mm distal to the nerve’s emergence from the globe (Bray et al., 2019). At various time points after crush, animals were humanely euthanized and tissues processed for further analyses.

### Immunohistochemistry

Mice were anaesthetized and transcardially perfused with 4% paraformaldehyde (PFA) in PBS. Tissues were harvested and postfixed in 4% PFA overnight at 4°C. For cryosectioning, optic nerve and eyes were cryoprotected in 30% sucrose in PBS for 48 h at 4°C before they were embedded in OCT compound (Tissue-Tek O.C.T.) and snap frozen in dry ice. Tissues were kept frozen at −80°C until ready to be sectioned at 10-μm thickness for the optic nerves and 16 μm for the retinas. Sections were immunostained by incubating in primary antibodies overnight at 4°C. Primary antibodies were diluted in PBS with 5% normal goat serum and 0.3% Triton X-100. Primary antibodies used were rabbit anti-protein S6 (pS6) diluted at 1:500 (Cell Signaling Technology, #2211), rabbit anti-RFP 1:1000 (Rockland, 600-401-379), rabbit anti-RNA-binding protein with multiple splicing (RBPMS) 1:200 (ProSci, 29-239), rabbit anti-GFAP 1:500 (Dako, Z033429) or chicken anti-GFAP 1:500 (Abcam, ab4674), and mouse anti-ITGAE 1:200 (Abcam, ab254182). Following primary antibody incubation, sections were washed and incubated in Goat Alexa Fluor IgG (H + L) secondary antibodies (Invitrogen, 1:500) at room temperature for 1 h. Following three washes with PBS, slides were mounted using Vectashield (Vector Laboratories).

### Imaging

Images were obtained using a Nikon Eclipse Ti fluorescent microscope or an Olympus FluoView 1000 (FV1000) confocal microscope. Confocal high-magnification 2D-projected optic nerve images were obtained by combining individual z-stacks images using the mosaic automated stitching mode of the Olympus FV1000 software. All images were analyzed with ImageJ (NIH).

### Quantification of retinal ganglion cell (RGC) survival and axon regeneration

The percentage of surviving RGCs in the injured retina was estimated by counting the number of RBPMS+ RGCs in several sections from the injured and the intact contralateral retina. RGC survival in the injured retina was presented as a percentage of the intact contralateral retina. To quantify the number of regenerating RGC axons, optic nerve sections were imaged, and the number of CTB+ axons at different distances distal to the lesion site was counted. Axon numbers are presented as number of axons per section normalized to 250-μm width. At least three to four sections were counted per animal.

### Quantification of pS6+ RGCs

For the determination of mammalian target of rapamycin complex 1 (mTORC1) activity in RGCs, several retina sections were immunostained with an antibody against the phosphorylated ribosomal pS6, a widely used marker for mTORC1 activity (Park et al., 2008; Yang et al., 2014; Wang et al., 2020). The number of pS6+ RGCs in the ganglion cell layer (GCL) of the injured retina was expressed as a percentage of the total number obtained in the contralateral intact retina.

### Lesion area quantifications

The area size (μm^2^) of the optic nerve lesion from different animals was determined by manually drawing a contour around the GFAP-negative area (lesion site) ∼1 mm away from the eye, using ImageJ software. At least three to four sections were analyzed for each animal. RT^2^ Profiler PCR Array. Total RNA was isolated from four mouse retinas for each condition using Direct-Zol RNA Miniprep (R2050; Zymo Research) followed by cDNA synthesis with the RT2 First Strand kit (330401; QIAGEN) according to manufacturer’s protocol. Real-Time PCRs for the RT2 Profiler PCR Arrays were performed using RT^2^ SYBR Green qPCR Mastermix (330501; QIAGEN), Mouse Extracellular Matrix & Adhesion Molecules Arrays in 96-well format (PAMM-013ZC; QIAGEN), and QuantStudio three Real-Time PCR System (Thermo Fisher Scientific). Analysis of exported data were conducted at QIAGEN’S GeneGlobe Data Analysis Center using a software-based tool (www.qiagen.com/shop/genes-and-pathways/dataanalysis-center-overview-page).

### Statistical analyses

Statistical analyses were performed using GraphPad Prism software. Data were analyzed using Student’s *t* test, one-way ANOVA, or two-way ANOVA with Tukey’s or Bonferroni’s *post hoc* test. Values of *p* < 0.05 were considered significant. Number of animals used for each animal group is stated in the figure legends. All error bars represent SEM.

## Results

### Most regenerating RGC axons associate closely with astrocytes

**Figure 1.**
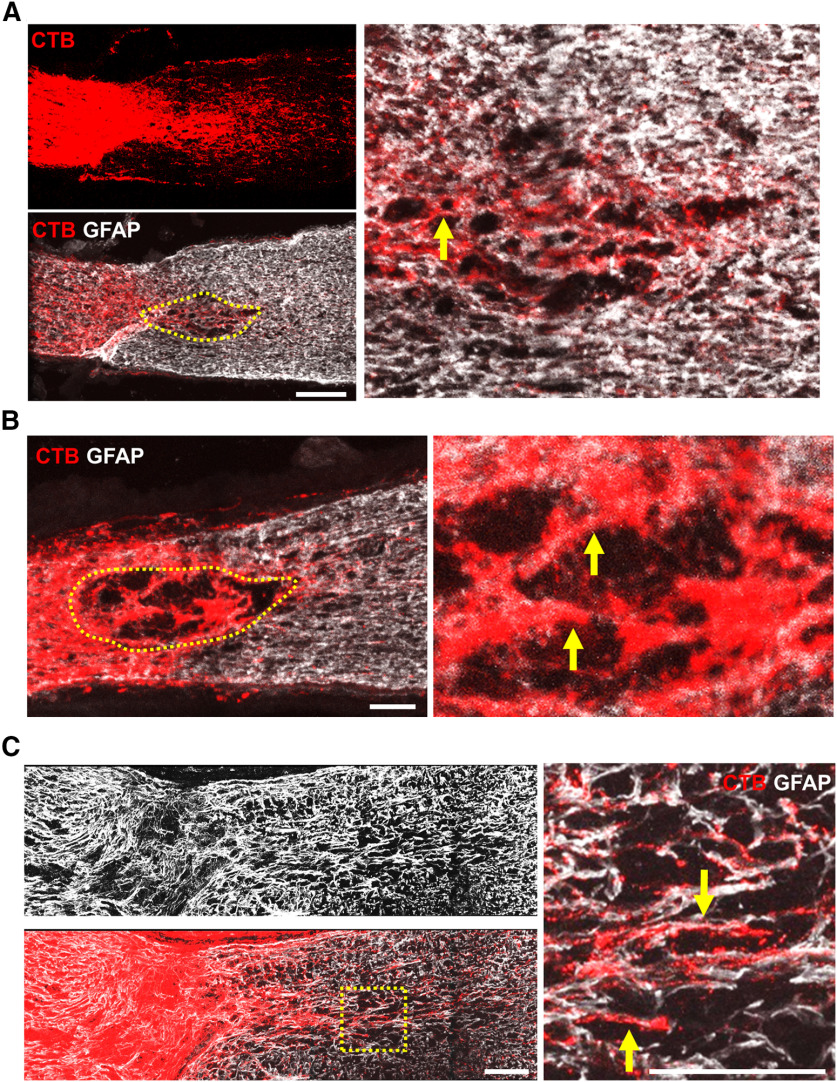
Regenerating RGC axons associate closely with GFAP+ processes. ***A***, Representative confocal image of an optic nerve section from an AAV-shPTEN-injected mouse three weeks after crush injury. Red, CTB555. White, GFAP immunoreactivity. Broken yellow line marks the boundary of the glial scar (i.e., lesioned area). Image on the right, higher magnification of the lesioned area. Yellow arrow points to an axon which appears to follow the GFAP+ processes through the lesion. ***B***, An optic nerve section from another AAV-shPTEN-treated mouse with a lesion larger than the animal shown in ***A***. Broken yellow line marks the boundary of the lesioned area. Image on the right, higher magnification of the lesioned area. Regenerating axons, indicated by the yellow arrowheads, seem to follow the GFAP+ processes through the lesion. ***C***, Representative confocal image of an optic nerve section from an AAV-CNTF-injected mouse three weeks after crush injury. Red, CTB555. White, GFAP immunoreactivity. Right panel, higher magnification of the boxed area in the left panel. The regenerating RGC axons (indicated by yellow arrows) appear to grow along the astrocyte processes. Scale bars, 100 μm.

To examine the roles played by astrocytes and cadherins in promoting RGC axon regeneration, we used intraorbital optic nerve crush in adult mice. To induce axon regeneration, we used shRNA against PTEN (shPTEN) to knock-down PTEN expression in adult RGCs. Adult mice received intravitreal injection of AAV-shPTEN, and two weeks later, animals received unilateral optic nerve crush. The optic nerves were assessed three weeks after crush. CTB was injected intravitreally 3 d before killing to anterogradely trace the regenerating RGC axons. Immunohistochemistry performed on the injured optic nerve using GFAP antibody showed the site of optic nerve crush. This is an area largely devoid of GFAP immunoreactivity, and located ∼1 mm from the eye. As expected, regenerating RGC axons were seen beyond the lesion site in the shPTEN-treated animals. Nearly all regenerating axons were found in close proximity to the GFAP+ processes in the lesion site, and they appeared to follow the astrocyte processes through the lesion (Fig. 1*A*,*B*). Similarly, regenerating axons immediately distal to the lesion site almost always associated closely with astrocyte processes. In addition to PTEN deletion, axon regeneration can also be stimulated by increasing CNTF expression (Müller et al., 2009; Yungher et al., 2015; Luo et al., 2016; Bray et al., 2019). Therefore, to determine whether axons associate with astrocyte processes under different types of regenerative conditions, we assessed the degree to which regenerating axons associate with astrocytes in AAV-CNTF-treated animals. To this end, adult mice received intravitreal AAV-CNTF injection, followed by optic nerve crush. Similar to the shPTEN-treated animals, the regenerating axons associated closely with the GFAP+ processes (Fig. 1*C*). Together, these results indicate that RGC axons under either condition regenerate into, and past, the lesion site along the astrocytic processes.

### Deleting NCAD in astrocytes decreases PTEN-knock-down-induced RGC axon regeneration

**Figure 2.**
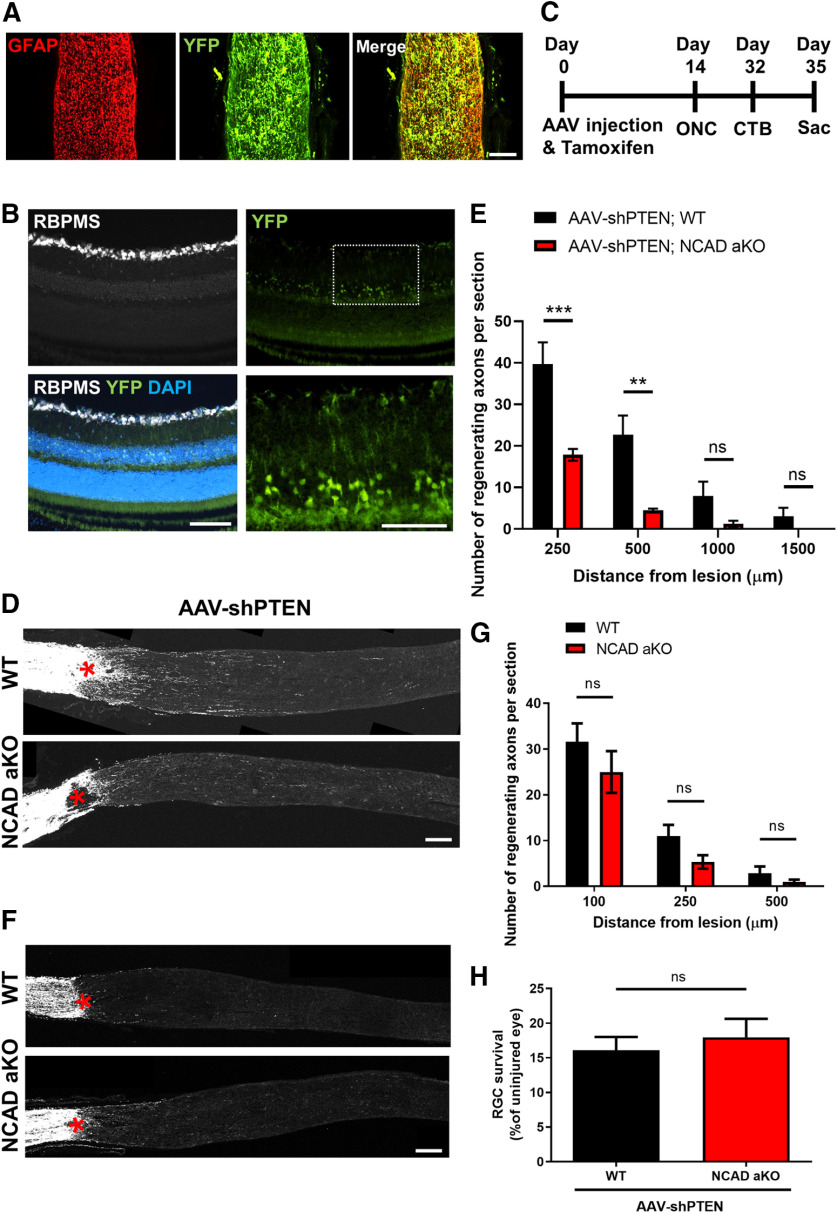
Deletion of NCAD in astrocytes prevents PTEN knock-down-induced axon regeneration in adult mice. ***A***, Cre recombination in the GFAPCreERT; Rosa26-YFP mouse. Representative optic nerve section from an adult GFAPCreERT; Rosa26-YFP mouse (eight weeks old) shows that most of the GFAP immunoreactivity is accompanied by YFP immunoreactivity, indicating high degree of Cre recombination in the astrocytes. Animal received five daily intraperitoneal injections of tamoxifen. Animals were perfused 2 d after the last tamoxifen injection. ***B***, Representative images of retinal sections (intact uninjured) of a GFAPCreERT; Rosa26-GFP mouse. Adult animals received tamoxifen injection for five consecutive days and perfused six weeks after the first tamoxifen injection. RBPMS in white, YFP in green, and DAPI in blue. YFP expression is predominantly in the inner nuclear layer. Some YFP immunoreactivity is also seen in the GCL. However, YFP immunoreactivity does not co-localize with RBPMS+ cells, indicating that there is no or minimal Cre expression in the RGCs. ***C***, Timeline of the astrocyte-specific NCAD KO experiment. ***D***, Representative optic nerve sections from AAV-shPTEN-injected WT (i.e., GFAPCreERT; YFP mice with tamoxifen injections) and AAV-shPTEN-injected astrocyte-specific KO mice (i.e., GFAPCreERT; YFP; NCAD^f/f^, or also referred in the figure as NCAD aKO) mice. Red asterisks, crush site. ***E***, Quantification of the number of regenerating axons per section at different distances from the lesion site. *N* = 5 mice for AAV-shPTEN; WT and *N* = 4 for AAV-shPTEN; NCAD aKO mice. Two-way ANOVA with Bonferroni’s *post hoc* test. ns, not significant; ∗∗*p* < 0.01, ∗∗∗*p* < 0.001. ***F***, Representative optic nerve sections from WT (i.e., GFAPCreERT; YFP mice with tamoxifen injections) and NCAD aKO mice without an AAV-shPTEN injection. Red asterisks, crush site. ***G***, Quantification of the number of regenerating axons for the WT and NCAD aKO groups without AAV-shPTEN injection. *N* = 4 for WT and *N* = 7 for NCAD aKO. Two-way ANOVA with Bonferroni’s *post hoc* test. ns, not significant. ***H***, Quantification of RGC survival for the AAV-shPTEN-injected WT and AAV-shPTEN-injected NCAD aKO mice shown in ***C***. *N* = 4 for each group. ns, not significant. Scale bars, 100 μm.

There are over 100 different types of cadherins found in vertebrates, which can be classified into four groups: classical, desmosomal, protocadherins, and unconventional (Maître and Heisenberg, 2013). NCAD belongs to the classical group and is found predominantly in neurons. To determine whether NCAD expression in astrocytes mediates RGC axon regeneration, we deleted NCAD specifically in adult astrocytes using the inducible Cre driver line, GFAP-CreERT (Casper et al., 2007). To first validate Cre recombination in the optic nerves of GFAP-CreERT mice, we crossed a GFAP-CreERT mouse to a Rosa26-loxp-stop-loxp-YFP reporter line (Rosa26-YFP) to generate GFAP-CreERT; Rosa26-YFP mice. Tamoxifen was administered daily for five consecutive days in these mice. Consistent with the previous reports, tamoxifen injection led to recombination in the majority of astrocytes; most GFAP immunoreactive cells co-expressed YFP (Fig. 2*A*). We did not quantify the percentage of astrocytes that express YFP. Nonetheless, previous reports have shown that ∼80–90% of astrocytes successfully express the transgene (Casper et al., 2007; Park et al., 2018). To confirm that Cre recombination does not occur in RGCs, we performed immunohistochemistry on the sectioned retinas of GFAPCreERT; Rosa26-YFP mice. We observed that the YFP+ cells were located predominantly in the inner nuclear layer where GFAP+ Müller cells are normally located (Fig. 2*B*). Some YFP immunoreactivity was also seen in the GCL, but these YFP+ cells did not co-localize with RBPMS immunoreactivity (i.e., a marker for RGCs), indicating that Cre recombination does not occur in RGCs. We crossed GFAPCreERT; Rosa26-YFP to NCAD^f/f^ mice to generate GFAP-CreERT; Rosa26-YFP; NCAD^f/f^ (hereafter referred to as GFAPCreERT; YFP; NCAD^f/f^). These animals received intravitreal AAV-shPTEN injection, followed by tamoxifen injection daily for five consecutive days. Two weeks after the AAV injection, animals received unilateral optic nerve crush. We injected CTB to label the regenerating axons (Fig. 2*C*). GFAPCreERT; YFP mice with the same treatment served as control animals (i.e., wild-type animals). Notably, we observed that axon regeneration was markedly reduced in the NCAD-deleted mice. There was approximately a 50% decrease in the number of regenerating axons at 250 μm distal to the lesion site (Fig. 2*D*,*E*). More distally, the reduction was even more obvious; in the NCAD-deleted mice, virtually no regenerating axons were seen beyond the 1000-μm mark. It is known that, even without regenerative treatment, a few RGC axons can regenerate spontaneously (Yin et al., 2006; Benowitz and Yin, 2010; Bray et al., 2019). To examine whether NCAD deletion has any effects on the spontaneous regeneration, we compared axon regeneration between the WT and NCAD-deleted animal groups, both without an AAV-shPTEN injection. There was no difference in the number of axons between these groups (Fig. 2*F*,*G*). These results demonstrate that, while astrocyte expression of NCAD is required for PTEN-knock-down-induced axon regeneration, it plays no role in promoting spontaneous RGC axon regeneration. Additionally, since the death of RGCs would affect axon regeneration, we also assessed RGC survival. Immunohistochemistry on retinas using an antibody against RBPMS (i.e., a marker for RGCs; Rodriguez et al., 2014) showed that RGC survival is unchanged after NCAD deletion in astrocytes (Fig. 2*H*).

### Deleting NCAD in RGCs decreases PTEN-deletion-induced axon regeneration

**Figure 3.**
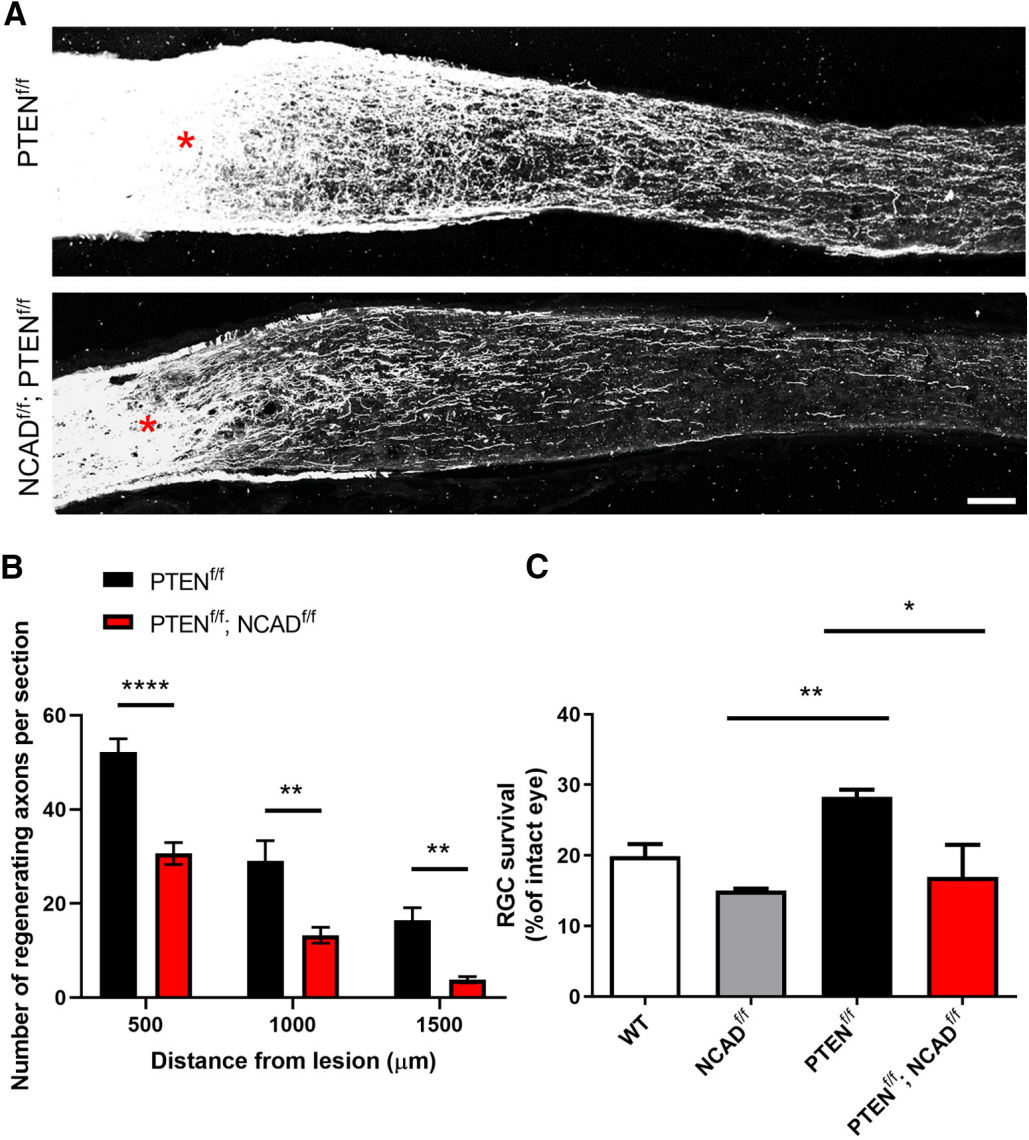
Deletion of NCAD in RGCs reduces PTEN deletion-induced axon regeneration. ***A***, Representative optic nerve sections from PTEN^f/f^ (i.e., single KO) and PTEN^f/f^; NCAD^f/f^ (i.e., double KO) mice. AAV-Cre was injected intravitreally two weeks before optic nerve crush. Red asterisks, crush site. ***B***, Quantification of the number of regenerating axons per section at different distances from the lesion site. *N* = 6 for PTEN^f/f^ and *N* = 14 for PTEN^f/f^; NCAD^f/f^. Two-way ANOVA with Bonferroni’s *post hoc* test; ∗∗*p* < 0.01, ∗∗∗∗*p* < 0.0001. ***C***, Quantification of RGC survival for the WT (i.e., C57BL/6J with AAV-Cre injection), NCAD^f/f^, PTEN^f/f^, and PTEN^f/f^; NCAD^f/f^ mice. One-way ANOVA with Tukey *post hoc* test; ∗*p* < 0.05, ∗∗*p* < 0.01. Scale bar, 100 μm.

The functionality of cadherins depends largely on the formation of homodimers. The homodimeric cadherins, including NCAD, create surface adhesion with cadherins present in the membranes of other cells, through changing conformation from *cis*-dimers to *trans*-dimers (Shapiro et al., 1995; Vendome et al., 2011). NCAD is expressed on apposing cell membranes of both axons and glial cells (Redies and Takeichi, 1996; Redies, 2000). In our previous study, we performed RNA-sequencing on murine RGCs and observed that *Cdh2* is highly expressed in at least two RGC subtypes (i.e., intrinsically-photosensitive RGCs and direction-selective RGCs; Bray et al., 2019). Other studies have reported that RGCs of different subtypes also express *cdh2* (Rheaume et al., 2018; Tran et al., 2019). Thus, we reasoned that NCAD is expressed on the RGC axons and promotes adhesion to astrocytes, thereby supporting axon regeneration. If this is true, then deleting NCAD in RGCs would reduce axon regeneration. To test this idea, we generated NCAD^f/f^; PTEN^f/f^ double floxed mice. In these mice, intravitreal injection of AAV2-Cre will result in deletion of both NCAD and PTEN in RGCs. Adult animals received intravitreal AAV2-Cre injection, followed by optic nerve crush two weeks later. The results showed that axon regeneration was drastically reduced in the NCAD^f/f^; PTEN^f/f^ double KO compared with the PTEN^f/f^ single KO mice (Fig. 3*A*,*B*).

### Deleting NCAD in RGCs eliminates PTEN-deletion-induced RGC survival

To assess whether deletion of NCAD in RGCs affects RGC survival, we performed immunohistochemistry using the RBPMS antibody. NCAD deletion alone had a statistically insignificant decrease in the RGC survival when compared with the WT mice. Previous studies have demonstrated that PTEN deletion in neurons promotes cell survival after injury or in neurodegenerative conditions (Park et al., 2008; Domanskyi et al., 2011). As shown in Figure 3, there was a difference in the number of surviving RGCs between the PTEN^f/f^ mice and the PTEN^f/f^; NCAD^f/f^ mice; three weeks after optic nerve crush, there was approximately a 50% reduction in RGC survival in the double KO mice (Fig. 3*C*) compared with the PTEN^f/f^ single KO mice.

### Deleting NCAD in astrocytes has no effect on CNTF-induced axon regeneration

**Figure 4.**
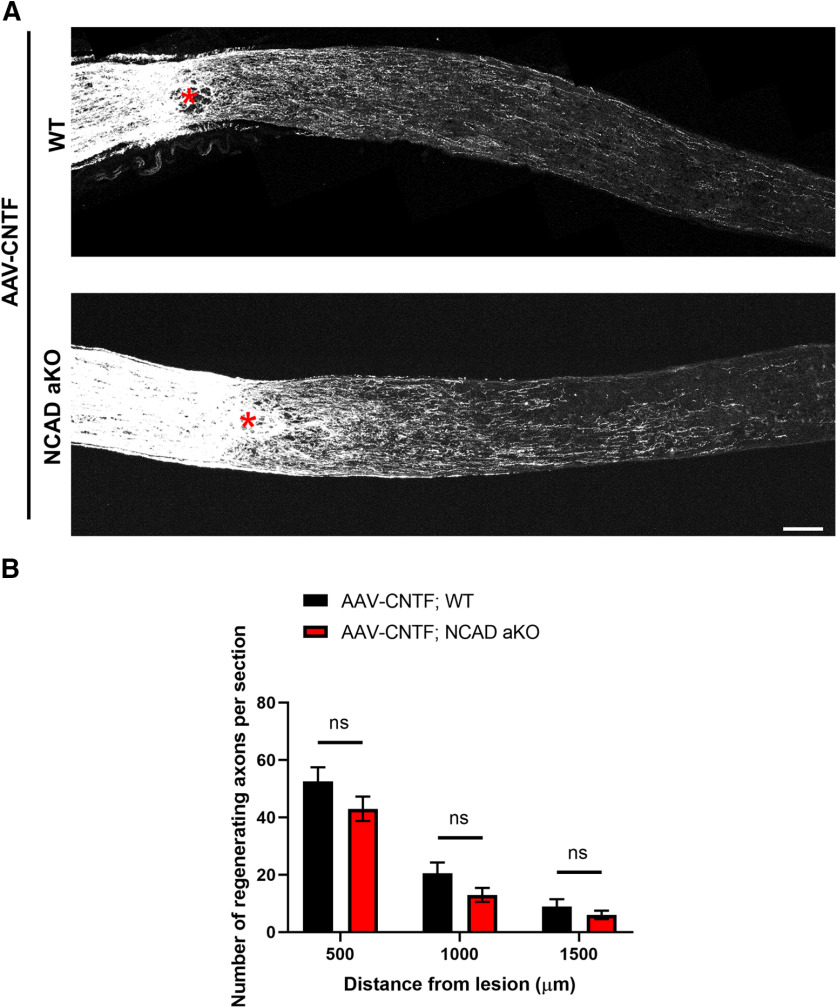
Deletion of NCAD in astrocytes does not significantly reduce CNTF-induced axon regeneration. ***A***, Representative optic nerve sections from AAV-CNTF-injected WT (i.e., GFAPCreERT; YFP) and AAV-CNTF-injected astrocyte-specific NCAD KO mice (i.e., GFAPCreERT; YFP; NCAD^f/f^ or also referred in the figure as NCAD aKO). Red asterisks, crush site. ***B***, Quantification of the number of regenerating axons per section at different distances from the lesion site. *N* = 7 for AAV-CNTF; WT and *N* = 13 for AAV-CNTF; NCAD aKO. Two-way ANOVA with Bonferroni’s *post hoc* test; ns, not significant. Scale bar, 100 μm.

Unlike PTEN, which regulates the activity of mTORC1 and glycogen synthase kinase 3 β (GSK3β; Park et al., 2008; Leibinger et al., 2019), CNTF promotes regeneration primarily through activation of STAT3 (Sun et al., 2011; Leibinger et al., 2013). To examine whether astrocytic NCAD plays a general role under different regenerative conditions, we assessed the effects of deleting NCAD in AAV-CNTF-treated animals. As expected, AAV-CNTF promoted axon regeneration in the GFAPCreERT; YFP (i.e., WT) mice. A similar level of CNTF-induced regeneration was seen in the GFAPCreERT; YFP; NCAD^f/f^ mice (Fig. 4*A*,*B*).

### Deleting NCAD in RGCs neither affects CNTF-induced axon regeneration nor RGC survival

**Figure 5.**
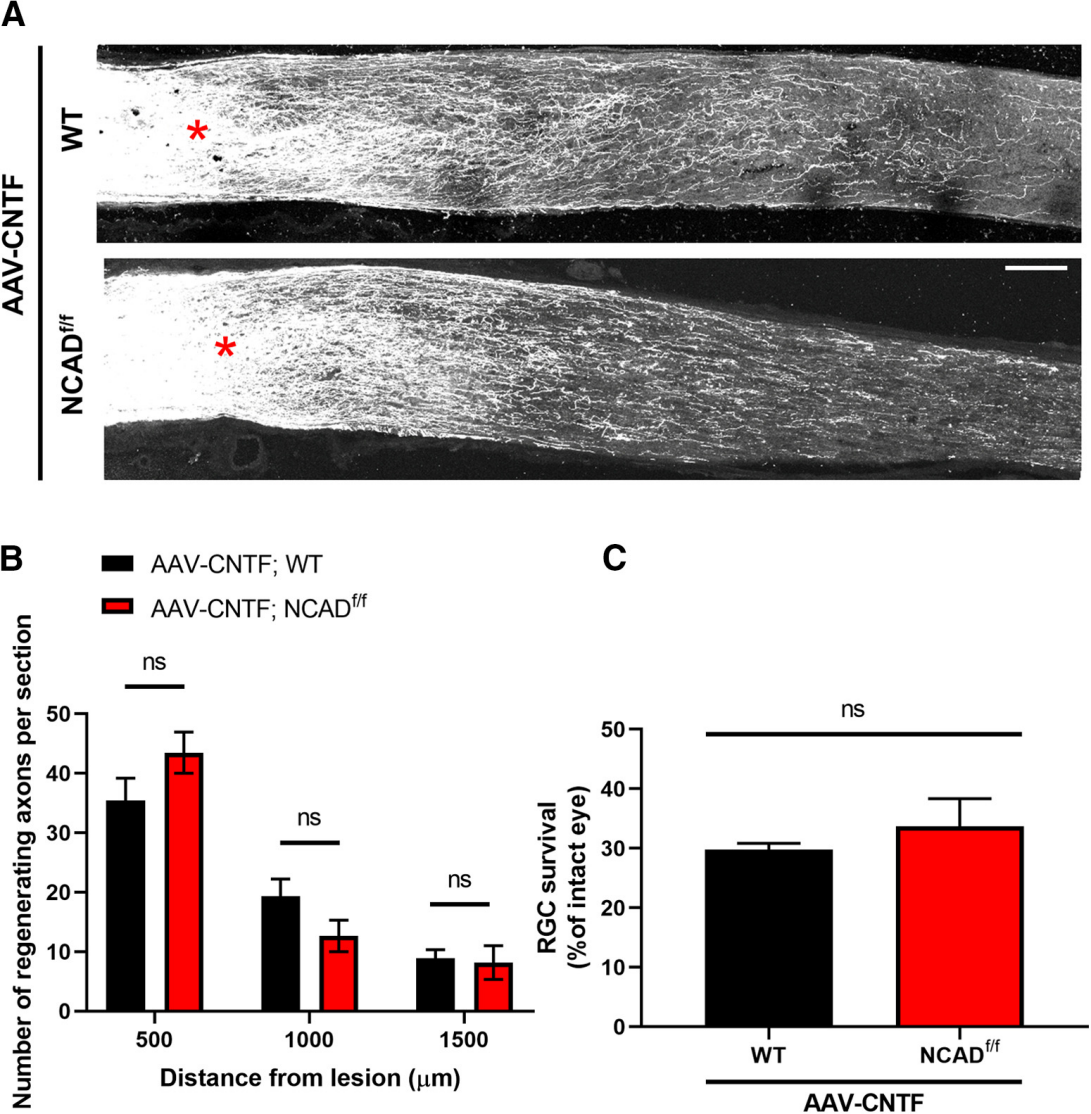
Deletion of NCAD in RGCs does not affect CNTF-induced axon regeneration or RGC survival. ***A***, Representative optic nerve sections from WT (C57BL/6J) and NCAD^f/f^ mice. AAV-Cre was injected intravitreally in both animal groups, followed by AAV-CNTF injection two weeks later. Animals received optic nerve crush 3 d after AAV-CNTF and survived for another three weeks after crush. Red asterisks, crush site. ***B***, Quantification of the number of regenerating axons per section at different distances from the lesion site. *N* = 9 mice/group. Two-way ANOVA with Bonferroni’s *post hoc* test; ns, not significant. ***C***, Quantification of RGC survival. Unpaired Student’s *t* test; ns, not significant. *N* = 5 mice/group; Scale bars, 100 μm.

As NCAD expression in RGCs was critical for axon regeneration and cell survival in the background of PTEN deletion, we examined whether NCAD in RGCs plays similar roles for CNTF-induced regeneration and RGC survival. NCAD^f/f^ mice received intravitreal AAV-Cre injection, followed by intravitreal AAV-CNTF injection two weeks later. Three days after the AAV-CNTF injection, animals received optic nerve crush. In contrast to the results seen in the PTEN-deleted animals, NCAD deletion in RGCs did not alter CNTF-induced regeneration or RGC survival (Fig. 5).

### Deleting NCAD in astrocytes does not affect the size of the lesion

**Figure 6.**
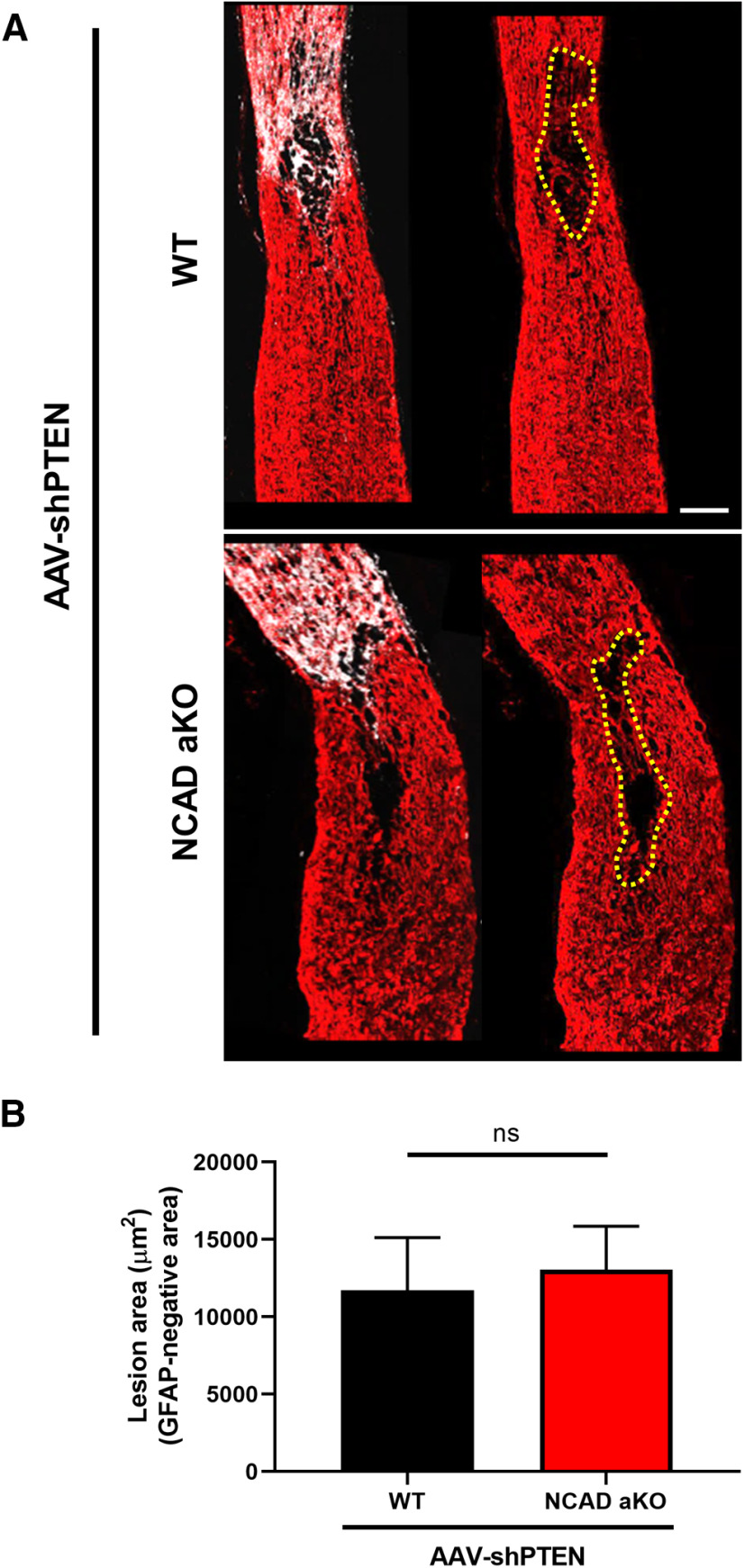
NCAD deletion in astrocytes does not alter the lesion size. ***A***, Representative optic nerve sections from AAV-shPTEN-injected WT (i.e., GFAPCreERT; YFP mice with tamoxifen injections) and AAV-shPTEN-injected astrocyte-specific KO mice (i.e., GFAPCreERT; YFP; NCAD^f/f^, or also referred in the figure as NCAD aKO) mice. Red, GFAP immunoreactivity; White, CTB-Alexa555. Broken yellow line marks the boundary of the glial scar (i.e., lesioned area). ***B***, Quantification of the lesion size. *N* = 5 for AAV-shPTEN; WT and *N* = 4 for AAV-shPTEN; NCAD aKO. Unpaired Student’s *t* test; ns, not significant. Scale bar, 100 μm.

Since the size of the lesion negatively correlates with axon regeneration, we also examined whether the deletion of NCAD in astrocytes increases the size of the lesion after optic nerve crush. Immunohistochemistry using GFAP antibody showed that the lesion size was unaffected by NCAD deletion in astrocytes (Fig. 6).

#### NCAD deletion in astrocytes does not change the level of mTORC1 activity in RGCs

**Figure 7.**
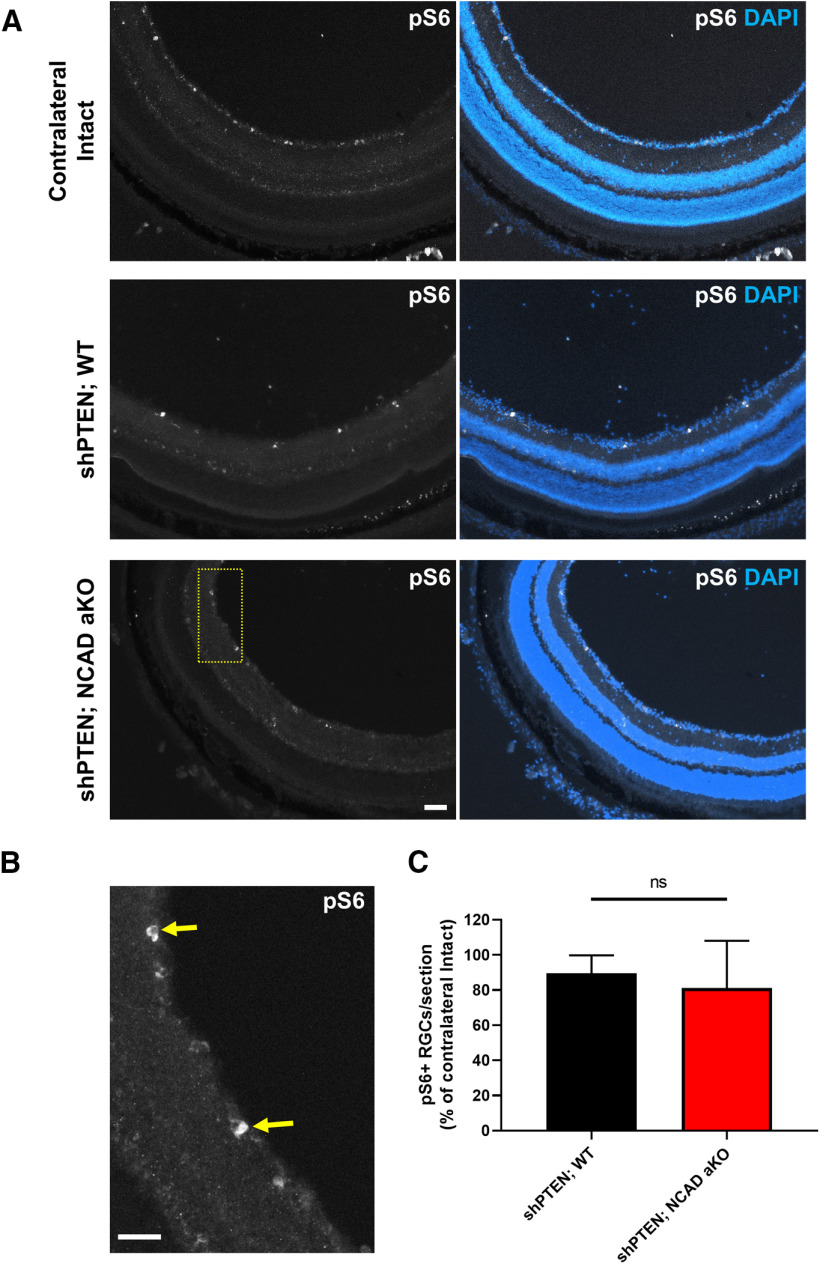
NCAD deletion in astrocytes does not alter the number of pS6 immunoreactive RGCs. ***A***, Representative images taken from retinal sections of contralateral intact, AAV-shPTEN-injected WT (i.e., GFAPCreERT; YFP mice with tamoxifen injections), and AAV-shPTEN-injected astrocyte-specific KO mice (i.e., GFAPCreERT; YFP; NCAD^f/f,^ or also referred in the figure as NCAD aKO) mice. Eyes were dissected out three weeks after optic nerve crush. ***B***, A higher magnification image of the boxed area in ***A***. Yellow arrows point to pS6+ RGCs. ***C***, Quantification of pS6+ RGCs, presented as percentage of the contralateral eye. Unpaired Student’s *t* test; ns, not significant. Scale bars, 50 μm.

Several studies have demonstrated that mTORC1 acts downstream of PTEN, and mediates axon regeneration (Park et al., 2008; Yang et al., 2014; Wang et al., 2020). To further examine the mechanisms by which NCAD deletion reduces axon regeneration, we assessed the level of mTORC1 activity in the RGCs of shPTEN-treated animals. To this end, we performed immunohistochemistry on sectioned retinas using an antibody against the phosphorylated ribosomal pS6, a known indicator of mTORC1 activity (Park et al., 2008; Yang et al., 2014; Wang et al., 2020). The numbers of pS6+ RGCs after injury were similar between the WT and NCAD-deleted animals (Fig. 7). Thus, these results indicate that the reduced regeneration and cell survival seen after NCAD deletion is unlikely to be caused by changes in mTORC1 activity.

#### Distinct CAMs are differentially expressed in the PTEN-deleted and CNTF-treated RGCs

**Figure 8.**
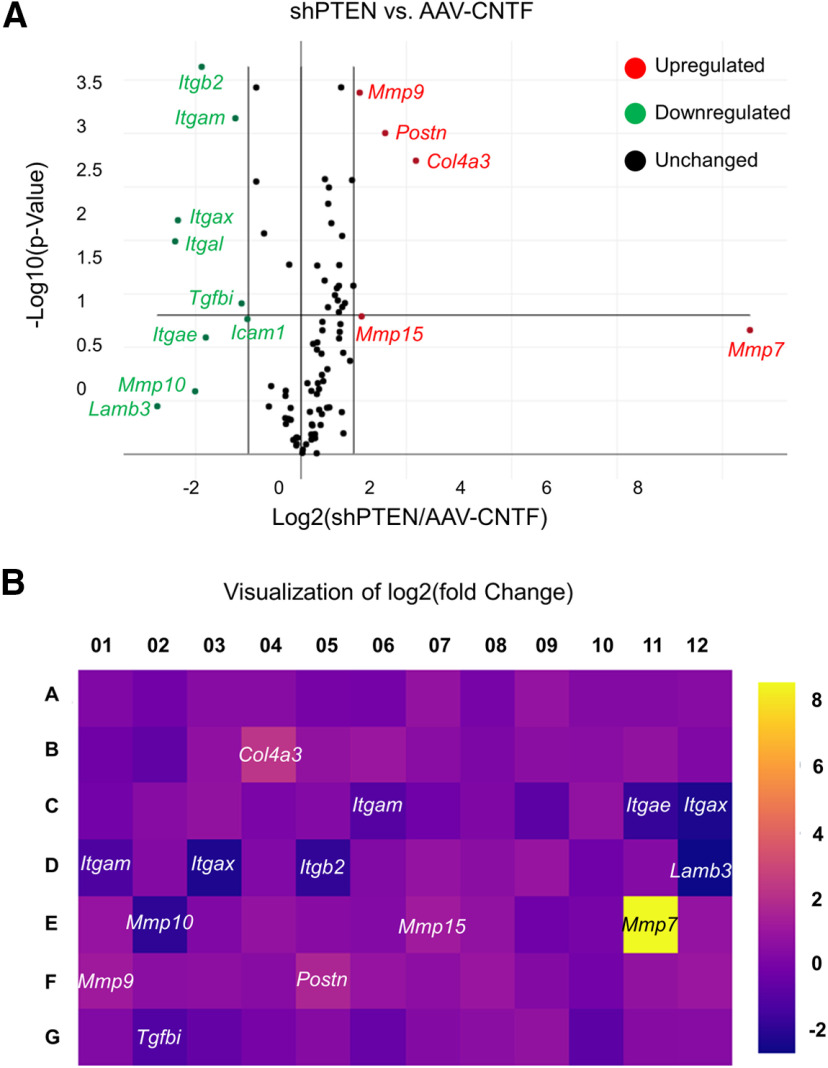
PCR array expression analysis shows differential expression of integrins between the shPTEN-treated and the CNTF-treated retinas. RT^2^ Profiler PCR Array was used to profile 84 genes on eight samples (i.e., four AAV-shPTEN-injected retinas and four AAV-CNTF-injected retinas). ***A***, The volcano plot identifies significant gene expression changes. Red circles, upregulated genes from shPTEN versus CNTF (i.e., genes expressed higher in shPTEN compared with CNTF). Green circle, downregulated genes from shPTEN versus CNTF (i.e., genes expressed lower in shPTEN compared with CNTF). Plotted are the log2 of the fold changes in gene expression on the *x*-axis versus their statistical significance on the *y*-axis. The center vertical line indicates unchanged gene expression, while the two outer vertical lines indicate the selected fold regulation threshold. The horizontal line indicates the selected *p* value threshold. ***B***, Heat map of the CAM and ECM RT^2^ Profiler PCR Array. The magnitude of the log2 fold change in mRNA expression of each gene is represented by the color of each square. Yellow indicates mRNA overexpression (i.e., genes expressed higher in shPTEN compared with CNTF). Purple indicates reduced mRNA expression (i.e., genes expressed lower in shPTEN compared with CNTF). Of the nine genes underexpressed in the shPTEN retinas (with fold regulation cut off of −2.0), five were integrin genes.

Our results show that NCAD is required for the PTEN-deletion induced but not for the CNTF-induced regeneration. What accounts for this difference? One possibility is that CAMs other than NCAD are induced in the CNTF-treated RGCs, and that these CAMs compensate for the loss of NCAD. This explanation is supported by the fact that (1) there are many different types of CAMs that can promote cell adhesion and growth, and that (2) cytokines are known to induce expression of various types of CAMs (Meager, 1999). To examine whether CNTF results in differential expression of CAMs in RGCs, we used RT^2^ Profiler PCR Array, and compared the expression of 84 CAMs and ECM genes (Table 1) between the AAV-CNTF-treated and the AAV-shPTEN-treated animals. Both groups received optic nerve crush 14 d after AAV injection, and retinas were removed 3 d after optic nerve crush. We extracted total RNAs from the whole retinas. As shown in Figure 8, several genes were differentially expressed between the two animal groups. Notably, nine genes were expressed at least two-fold higher in the CNTF animals. Of these 9, 5 were integrins, namely *Itgae*, *Itgal*, *Itgam*, *Itgax*, and *Itgb2*. On the other hand, there were five genes expressed at least two-fold higher in the PTEN-deletion animals, namely *Col4a3*, *Mmp15*, *Mmp7*, *Mmp9*, and *Postn* (Fig. 8).

**Table 1:** The array layout in **Figure 8, the gene symbol and value of log2 fold change of mRNA expression (shPTEN compared with CNTF)**

Layout	1	2	3	4	5	6	7	8	9	10	11	12
A	Adamts1/1.14	Adamts2/−1.18	Adamts5/1.32	Adamts8/1.31	Cd44/−1.06	Cdh1/−1.11	Cdh2/1.68	Cdh3/−1.06	Cdh4/1.72	Cntn/1.23	Co1aa/1.23	Col2a1/1.32
B	Col3a1/−1.23	Col4a1/−1.63	Col4a2/1.56	Col4a3/4.53	Col5a1/1.65	Col6a1/1.99	Ctgf/1.37	Ctnna1/1.07	Ctnna2/1.44	Ctnnb1/1.36	Ecm1/1.66	Emillim1/1.15
C	Entpd1/−1.15	Fbln1/0.129	Fn1/1.65	Hapln1/1.02	Hc/1.2	Icam1/−2.03	Itga2/−1.22	Itga3/1.12	Itga4/−1.8	Itga5/1.62	Itgae/−3.50	Itgal/−5.24
D	Itgam/−2.37	Itgav/1.23	Itgax/−5.05	Itgb1/1.15	Itgb2/−3.7	Itgb3/1.16	Itgb4/1.72	Lama1/1,41	Lama2/1.78	Lama3/−1.22	Lamb2/1.25	Lamb3/−6.63
E	Lamc1/1.74	Mmp10/−4.03	Mmp11/1.14	Mmp12/1.7	Mmp13/1.34	Mmp14/1.2	Mmp15/2.22	Mmp1a/1.6	Mmp2/−1.23	Mmp3/−1.05	Mmp7/367.49	Mmp8/1.71
F	Mmp9/2.16	Ncam1/1.41	Ncam2/1.49	Pecam1/1.31	Postn/3.03	Sele/1.75	Sell/1.45	Selp/1.91	Sgce/1.23	Sparc/−1.15	Spock1/1.65	Spp1/1.96
G	Syt1/1.17	Tgfbi/−2.19	Thbs1/−1.53	Thbs2/−1.06	Thbs3/1.27	Timp1/−1.48	Timp2/1.24	Timp3/1.43	Tnc/1.64	Vcam1/−1.8	Vcan/1.27	Vtn/1.32

**Figure 9.**
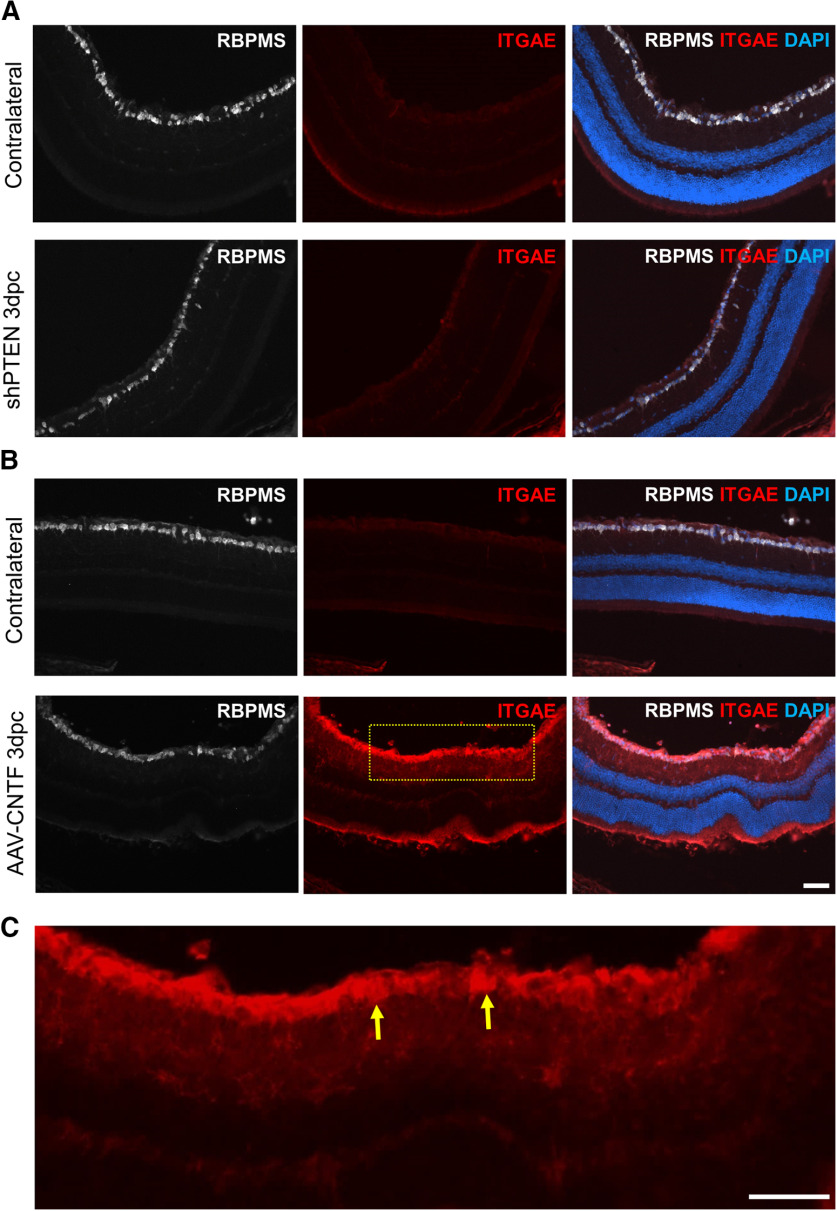
Differential expression of ITGAE in the retinas of AAV-CNTF-treated and AAV-shPTEN-treated mice. ***A***, Representative images taken from retinal sections of the AAV-shPTEN-injected and the contralateral intact eyes. Sections were immunostained with antibodies against RBPMS (white) and ITGAE (red). ***B***, Representative images taken from retinal sections of the AAV-CNTF-injected and the contralateral intact eyes. Eyes were dissected out 3 d postcrush (3 dpc). Similar results were seen in two independent animals from each AAV group. ***C***, A higher magnification image of the boxed area in ***B***. Yellow arrows point to ITGAE immunoreactive RGCs. Scale bars, 50 μm.

Integrins play key roles in promoting cell adhesion and neurite growth (Tomaselli et al., 1988; Izumi et al., 2017; Nieuwenhuis et al., 2018). High expression of integrins in the CNTF-treated animals supports the notion that the axons in these animals may use integrins for regeneration. To determine whether integrins are, in fact, expressed higher in the CNTF-treated RGCs, we performed immunohistochemistry on sectioned retinas. Animals received either AAV-CNTF or AAV-shPTEN injection, followed by crush two weeks later. Three days after crush, animals were perfused and eyes processed for cryosection. We tested antibodies against ITGAX and ITGAE. These antibodies were selected because several studies in the past successfully used these antibodies for immunohistochemistry. However, the ITGAX antibody failed to show obvious difference in the immunoreactive signals between the two animal groups (data not shown). On the other hand, we observed a striking difference in the intensity of ITGAE immunoreactivity between the two treatment groups. In the CNTF-treated RGCs, intense ITGAE immunoreactivity was observed, while the signals in the PTEN-deleted or uninjured RGCs were very weak (Fig. 9). We also observed that ITGAE expression was higher in layers other than the GCL, indicating that CNTF results in induction of ITGAE not only in RGCs, but also in other retinal cell types. Overall, these results show that various integrins were highly expressed after CNTF treatment, which could explain why NCAD deletion had a minimal effect on axon regeneration in the CNTF-treated animals.

## Discussion

Despite the decades’ long demonstration of lengthy CNS axon regeneration, our knowledge of the glial factors that support the migration of these axons after injury is rather limited. We do know that, depending on the types of regenerative stimuli, some axons appear to be able to migrate successfully toward their targets. In many cases, however, axons take circuitous routes back toward the cell body (Luo et al., 2013; Pernet and Schwab, 2014; Yang et al., 2020). As functional regeneration requires innervation of axons to their correct targets, we are becoming increasingly aware of the importance of defining the cellular and molecular factors that regulate this process in adulthood.

Axons cannot simply elongate in space. Rather, they normally bind to substrates. Several studies have demonstrated that regenerating axons follow astrocytes. In the mature CNS, there are two main types of astrocytes, the protoplasmic and the fibrous. The former resides mainly in the gray matter, whereas the latter resides in the white matter. Thus, in the optic nerve, which is a white matter tract, the astrocytes consist predominantly of the fibrous type. These fibrous astrocytes are further classified into three subtypes based on their morphology: the transverse subtype, with processes projecting mostly perpendicular to the optic nerve; the longitudinal subtype, with processes projecting mostly parallel to the optic nerve; and the random subtype, with processes projecting in both directions (Butt et al., 1994). These subtypes make up ∼34%, 48%, and 18% of the total optic nerve astrocytes in mice, respectively (Butt et al., 1994). It is quite possible that regenerating RGC axons simply follow whichever astrocytes they come in contact with. If this is the case, then these axons will travel stochastically in all different directions, perpendicular or in parallel to the optic nerve. In support of this notion, our studies and others have shown that, in the case of regenerative treatments involving CNTF, many RGC axons in the optic nerve meander in all different directions (Luo et al., 2013; Pernet et al., 2013; Pernet and Schwab, 2014). Others have shown that some RGCs can grow toward the brain more effectively under different regenerative conditions (de Lima et al., 2012; Lim et al., 2016).

In addition to the astrocytes, other environmental factors can affect the direction of RGC axons, including immune cells, oligodendrocytes, myelin, and various growth factors and ECM proteins secreted by the glial cells (Duffy et al., 2012; Fawcett et al., 2012; Sharma et al., 2012; Geoffroy and Zheng, 2014). The myelin and axons distal to the injury site undergo Wallerian degeneration. Macrophages and, to some extent, the local microglial cells migrate throughout the injured nerve, and engulf the degenerated residues. The myelin debris and the immune cells not only physically affect the path of axons, but they also affect axon trajectories through their expression of various repulsive molecules on their surfaces, including Nogo, myelin associated glycoprotein, oligodendrocyte myelin glycoprotein, and PirB (Atwal et al., 2008; Pernet and Schwab, 2012; McKerracher and Rosen, 2015).

In this study, we focused on examining NCAD’s roles on axon regeneration *in vivo*. It has long been known that one of NCAD’s primary functions in the CNS is promoting cell-cell adhesion. In line with this function, deleting NCAD in astrocytes decreased axon regeneration, at least in the background of PTEN deletion. While it is likely that the loss of regeneration is because of the loss of NCAD’s homophilic adhesive action in the KO mice, we cannot rule out the possibilities that other mechanisms contributed to this loss. The activity of mTORC1 in RGCs is an important neuron intrinsic factor for RGC axon regeneration after PTEN deletion. However, NCAD deletion did not affect mTORC1 activity, which suggests that this mechanism is not involved. It remains to be seen whether NCAD controls other signaling pathways in RGCs relevant to axon regeneration, including KLFs and HDACs. It seems unlikely that NCAD deletion in astrocytes will cause changes in neuron-intrinsic signaling factors, but nonetheless, this possibility has not been examined.

A previous study has shown that blocking NCAD functions in the spinal cord using an NCAD antibody reduces the formation of inhibitory fibrotic scar, thus resulting in enhanced axon regeneration (Hara et al., 2017). It has also widely been shown that lesion size negatively correlates with axon regeneration (i.e., the larger the lesion, the smaller degree of axon regeneration). In our model, where NCAD was deleted specifically in astrocytes, we did not observe obvious changes in lesion size. However, NCAD deletion could have altered the lesion environment in different ways, and caused a decrease in regeneration. For instance, astrocytes are known to secret various regeneration promoting factors, including cytokines and trophic factors (Wiese et al., 2012; Anderson et al., 2016). Thus, another possibility is that NCAD deletion may have reduced the release of such factors in the injured optic nerve. Alternatively, considering that NCAD would have been deleted in the astrocytes and the Müller cells in the retinas of GFAPCreERT; NCAD^f/f^ mice, it is also possible that the reduced regeneration may have resulted from changes that took place in these cells locally in the retina.

It is also worth noting that several studies have described the existence of multiple transcript variants of NCAD resulting from alternative splicing. In fact, the expression patterns of distinct NCAD isoforms and their roles have been studied extensively in the invertebrate species including the *Drosophila* (Yonekura et al., 2006; Schwabe et al., 2014). *In vitro* cell aggregation assays performed on *Drosophila* cell lines revealed that all NCAD isoforms mediate homophilic interactions, but the isoforms encoded by different exons exhibit different adhesive activity (Yonekura et al., 2006). These studies have suggested that NCAD alternative splicing might provide a mechanism to fine-tune its adhesive activity at different developmental stages. However, the extent to which different NCAD isoforms are expressed in the adult mice, and whether they contribute to RGC axon regeneration and neuronal survival in the mammals remain unknown.

Integrins are α/β heterodimeric adhesion glycoprotein receptors that regulate critical cellular processes including cell adhesion, and growth. Integrins bind ECM proteins through their ectodomain, and the presence of an appropriate integrin heterodimer on the neuronal surface determines whether a regenerating neuron possesses the ability to grow on a particular ECM molecule (Tan et al., 2011; Eva and Fawcett, 2014; Fawcett, 2017). Integrins are known to interact with many astrocyte-expressed factors that can directly or indirectly support cell adhesion. For example, osteopontin is recognized by α4β1, α5β1, αVβ1 and αVβ5, while tenascin-C is recognized by α8β1 and αVβ3. Moreover, integrin-αVβ3 forms complexes with syndecan-4, which in turn interacts with neuronal Thy-1 receptors (Avalos et al., 2009; Hillen et al., 2018). Moreover, *in vitro* studies have demonstrated that, in addition to NCAD, integrins are a major surface system that mediates neurite outgrowth on astrocyte surfaces; NCAD functions prominently in the outgrowth of neurites on astrocytes by E8 and E14 chick ciliary ganglion (CC) neurons. NCAD and integrin β1 antibodies together eliminates CG neurite outgrowth on cultured astrocytes. Thus, it was proposed that NCAD and integrin receptor systems are important in regulating axon growth on astroglia *in vivo* (Tomaselli et al., 1988).

In contrast to the results seen in the PTEN-deleted mice, we observed that NCAD deletion, either in RGCs or astrocytes, does not reduce CNTF-induced regeneration. These results suggest that the manner by which regenerating axons adhere to glial cells might be quite distinct for axons under different conditions. Since different combinations of integrin heterodimers can promote adhesion to astrocytes, and thus they have the ability to compensate for NCAD deletion, integrins may have seized a dominant role and propelled axon regeneration in the CNTF-treated animals. Furthermore, our studies and others support a model in which CNTF overexpression leading to induction of differential expression of integrins (and possibly other CAMs) allows axons to adhere on the various astrocyte subtypes more extensively. This, in turn, will result in circuitous axon growth in the injured optic nerve. In our unpublished work, we applied 3D imaging and traced single regenerated RGC axons (Bray et al., 2017) in PTEN-deleted mice. In these mice, we observed less aberrant RGC growth (i.e., transverse and circular growth) in the optic nerve compared with the AAV-CNTF-injected mice, supporting the proposed model. Having said that, we did not functionally examine whether the various integrins expressed highly in CNTF animals in fact mediate RGC axon adhesion to astrocytes and promote regeneration. Furthermore, as mentioned above, numerous factors can affect the path of axons. Thus, it remains rather speculative that differential expression of CAMs in RGCs causes the various growth patterns seen under different regenerative conditions.

Under different regenerative conditions, it is also possible that CAMs and guidance factors are differently expressed in the astrocytes (or in other cell types) in the injured optic nerve, thus contributing to the difference seen between the PTEN deleted and the CNTF-treated animals. In a previous study, RNA-sequencing performed on the spinal cord astrocytes after spinal cord injury revealed changes in the expression of several genes whose products are known to mediate cell adhesion or axon-glial interaction (Anderson et al., 2016). For example, Anderson et al., has shown that the expression of *Cntn2*, *Itgam*, *Itgb2*, *Ephb1*, and *Vcan* were upregulated more than twofold in the astrocytes after spinal cord injury. On the other hand, there was ∼40% reduction in the expression of *Cdh2* in the astrocytes after SCI (Anderson et al., 2016). In an unpublished study, we performed RNA-sequencing on the optic nerve astrocytes of adult mice. We observed that the expression of *Cdh2* in the optic nerve astrocytes is unchanged after optic nerve crush. Nonetheless, we observed that numerous genes that encode CAMs and guidance factors are differentially expressed in the optic nerve astrocytes after injury. For example, *Cdh15*, *Icam1*, *Sdc4*, and *Epha3* were upregulated more than twofold after optic nerve crush injury (unpublished observation). However, it remains to be seen whether the products of these genes in fact modulate the glial environment at the lesion site, or mediate axon-glial interaction, and promote RGC axon regeneration.

Cadherin-mediated cell-cell adhesions have been implicated in regulating cell survival during development and preserving tissue homeostasis. Indeed, studies have reported that interfering with cadherin adhesion triggers apoptosis in various non-neuronal cells, including tumor cells (Nguyen et al., 2018). However, NCAD’s specific role for neuronal survival is not well understood. One *in vitro* study reported that NCAD contributes to the survival of dissociated neurons (Lelièvre et al., 2012); plating spinal or hippocampal neurons on NCAD recombinant substrate enhanced neuronal survival compared with non-specific adhesion on poly-L lysine. NCAD engagement, in the absence of other survival factors (cell-matrix interactions and serum) was shown to protect neuronal cells against apoptosis. Nonetheless, to our knowledge, our study is the first to demonstrate that NCAD contributes to the survival of CNS neurons after injury. The mechanism by which NCAD provides neuroprotection is unclear. The *in vitro* study did examine the potential signaling pathways involved; the phosphoinositide 3-kinase (PI3K)/Akt survival pathway and its downstream effector BAD were not involved in the NCAD-mediated survival of GT1-7 neuronal cells. In contrast, NCAD activated the Erk1/2 MAP kinase pathway, reducing the level of the proapoptotic protein Bim-extralong (EL) in these cells (Lelièvre et al., 2012). Another study using a prostate carcinoma line demonstrated that NCAD-catenin adhesion complex results in up-regulation of the anti-apoptotic protein Bcl-2, whereas the level of the proapoptotic protein Bax remained constant. NCAD homophilic ligation initiated PI3K-dependent activation of Akt resulting in Akt phosphorylation of Bad (Tran et al., 2002). Together, these studies indicate that interruption of NCAD-mediated cell-cell interactions can induce an increase in the expression of the proapoptotic Bcl-2 family of proteins and tip the balance toward programmed cell death. If so, it is unclear why NCAD deletion affected RGC survival in the PTEN-deleted, but not in the CNTF-treated animals. It seems plausible, however, that CNTF induces signaling pathways, including the JAK/STAT3 pathway that can counterbalance the detrimental effects of NCAD deletion.

Several studies have documented that different RGC subtypes respond differently to an injury with some RGC subtypes more resilient than others (Duan et al., 2015; Bray et al., 2019; Tran et al., 2019). We note that the AAV-Cre used in this study deletes NCAD in whole RGCs, and not in specific RGC subtypes. Our results showed that NCAD deletion in RGCs reduces the overall number of surviving RGCs after PTEN deletion. However, some RGC subtypes may express NCAD and rely on this protein for survival and/or regeneration while other subtypes might be NCAD independent. Alternatively, NCAD may in fact function in a detrimental manner in some RGC subtypes after injury, and deletion of NCAD in these RGCs may have actually resulted in increase in their survival and regeneration. In this sense, defining NCAD’s general role in the CNS neurons using the AAV-Cre approach is not without a caveat. While it is beyond the scope of this current study, it might be interesting in the future to use Cre driver lines that allow deletion of NCAD in specific RGC subtypes, and comprehensively determine NCAD’s role in RGCs.

In summary, we show that NCAD can engage in promoting the survival of CNS neurons and axon regeneration. However, its contributions are dispensable, and likely compensated for by other factors under certain stimulating conditions. The last decade has unveiled multiple strategies that promote lengthy axon regeneration. However, the propelled growth is accompanied by a lack of direction in the degenerated tract, warranting investigations into the cellular and molecular guidance factors that determine the fate of growing axons. Dissecting the mechanisms by which NCAD and other CAMs shape the paths of axons could help devise better ways to promote functional axon regeneration.

## Synthesis

Reviewing Editor: Sunghee Cho, Burke Neurological Institute/Feil Family Brain and Mind Research Institute, Weill Cornell Medicine Decisions are customarily a result of the Reviewing Editor and the peer reviewers coming together and discussing their recommendations until a consensus is reached. When revisions are invited, a fact-based synthesis statement explaining their decision and outlining what is needed to prepare a revision will be listed below. The following reviewer(s) agreed to reveal their identity: Xin Duan. Note: If this manuscript was transferred from JNeurosci and a decision was made to accept the manuscript without peer review, a brief statement to this effect will instead be what is listed below.

The presented study, addressing a role for neural cadherin in optic nerve regeneration, has been evaluated by two reviewers who have expertise in the field. Both reviewers concurred on the strength of the study, noting that the presented data is convincing, the study design is comprehensive in detailing the cell-type specific role of neural cadherin, and the information contributes to advance the axon regeneration field. However, the reviewers also brought several points that require clarification and/or some additional data. These include the regulation of CDH2 expression, changes of adhesion and guidance molecules following the injury, and RGC subtype specificity that could lead to insights on the observed difference between the two regenerative conditions. The detailed reviews and specific comments are appended below.

Reviewer 1

During development, growing axons need to navigate over long distance to reach appropriate targets. After injury to the adult mammalian central nervous system, the very same axons need to cover a much longer distance to re-innervate appropriate targets. Strategies that promote long distance axon regeneration have been recently developed. However, only few regenerating axons are effectively guided towards appropriate target areas, negatively impacting functional connectivity and neurological recovery. After Pten silencing and genetic deletion, the authors of this study have found that the vast majority of regenerating RGC axons closely associate with GFAP+ processes at the lesion site. During development of the nervous system, growing axons are guided by cell adhesion molecules expressed on the surface of glial cells or neurons. Of the different types of cadherins found in vertebrates, the authors focused on NCAD, a classical cadherin and member of the cadherin superfamily that is predominantly expressed in neurons. Interestingly, axon regeneration is significantly reduced in NCAD deleted mice with no regenerating axons found at the 1 mm mark. Whereas astrocyte expression of NCAD is required for PTEN-knockdown induced axon regeneration, the authors found NCAD plays no role in promoting RGC axon regeneration upon CNTF stimulation. The authors then used a PCR array to examine differential gene expression of integrins known to facilitate cell adhesion in Pten silenced and CNTF-treated retinas. I found these results novel and convincing. The manuscript is well written and the figures are curated with attention to details. The data will definitely stimulate further discussion about the role of astrocytes in axon regeneration.

Here are my minor comments for improvement.

-How is CDH2 expression regulated in the injured optic nerve?

-What are the gene expression changes of cell adhesion and guidance molecules in injured optic nerve astrocytes? In addition to CDH2, do the authors see any change in genes known to mediate axon-glial interaction? The authors may consider adding a few lines in the discussion about CDH2 alternative splicing as this results in multiple transcript variants that may impact on the ability to regenerate axons.

- Appropriate reference/s needed for the following sentence “CAMs are a large group of proteins located on the cell surface, involved in the binding of cells to glial cells or extracellular matrix(ECM) proteins.” (Page 4, lines 73-75).

- Page 16 line 387 “Prototype” should be changed with “protoplasmic"

- “7regeneration” should be corrected (Page 32, Line 751)

- Scale bars are missing in Figures 1A, 2C

- Figure 8A: the authors may consider changing the color of the gene name (red or green) to match the color of each representative dot.

Reviewer 2

In this manuscript, the authors carried out an excellent and comprehensive study. They investigated the role of cell neural cadherin (NCAD) in astrocytes, the different effects of deletion of NCAD in RGCs between axon regeneration and cell survival, and distinct CADs control axon regeneration in different regenerative conditions. The work comes timely to link the cell-cell recognition to regeneration, which is a timely work for the field of optic nerve regeneration. The link from cell-cell recognition to the integrin pathway offers future insights into this pathway in vivo, esp. in terms of future therapeutics.

In the axon regeneration field, the internal and external conditions determine the axon regeneration rate. One of the most important external factors that determine axon regeneration is the glial cells that may provide substrates for axon to regenerate. However, the role of glial CADs to determine the axon regeneration is unclear. The authors use GFAP-CreER mice to knock out the CADs in astrocytes and observe the anatomical, morphology, and molecular change in two different conditions. The results are precise and experimental design is systematical. This manuscript gives us a new sight to study the glial cells in axon regeneration, especially the molecular mechanism of the CADs in glial cells for axon regeneration.

Another critical question the authors addressed is that after optic nerve crush, how the whole cells change, such as the axon degenerate and the cell soma death. This manuscript compares these conditions after using the AAV2-Cre virus to delete the NCAD in RGCs. They found that deleting NCAD in RGCs decreased axon regeneration and eliminated PTEN-deletion induced RGCs survival. However, the authors only provide us some anatomy and cell number change results, more details such as cell-type specificity and molecular mechanism are necessary.

For the final part, they compare the difference between two regenerative conditions; one is PTEN deletion; another is increasing CNTF expression. For these two regenerative conditions, only the lesion site along the astrocytic processes experiment has similar results, in other experiments, such as CADs knock out in astrocytes, and in RGCs, these two conditions have the different phenotype. In order to solve these problems, the authors used RT2 Profiler PCR Array to compare the expression of 84 CADs and ECM genes; they found several genes that are differentially expressed between these two conditions and provide us some immunohistochemistry results to confirm their explanation.

As a study of CAD in axon regeneration and cell survival, the manuscript extends our current study and suitable for the readers of eNeuro. However, if the authors can provide us more details about the molecular mechanism underline the process, the readers should form a system knowledge network.

By examining the data presented here, I have several critiques for the authors to address.

Major Critiques:

1) The leakage of the GFAP-CreER mice: The authors used GFAP-CreER mice to specifically deletion CADs in astrocytes. However, the GFAP also expressed in RGCs according to other reports. The authors should check the leakage of the GFAP, provide readers some evidence of the expressed explicitly in astrocytes, or at least discuss this issue more precisely.

2) Cell types specificity in the RGCs NCAMs deletion experiments: in their previous study, they used RNA-seq to find NCAD is highly expressed in at least two RGC subtypes (Duan et al., 2015; Bray et al., 2019). Different cell types have different responses to axon regeneration and survival. The authors use AAV2-Cre to delete the NCAD in whole RGCs; this manipulation may lead to a mix of different cell subtypes phenomenon. These also may lead to the difference between the axon regeneration and cell survival; if the author focuses on one single subtype of RGCs, they should get more definite results. Moreover, this is also an explanation for the difference between the PTEN deletion and CNTF overexpression.

Minor points:

1) In Figure 1, the arrangement of the images is misleading. Figure 1B should be put into a supplementary figure. Figure 1C should have the same images and layout as Figure 1A; it is easier for readers to compare the PTEN deletion and CNTF overexpression results.

## References

[B1] Anderson MA, Burda JE, Ren Y, Ao Y, O'Shea TM, Kawaguchi R, Coppola G, Khakh BS, Deming TJ, Sofroniew MV (2016) Astrocyte scar formation aids central nervous system axon regeneration. Nature 532:195–200. 10.1038/nature17623 27027288 PMC5243141

[B2] Anderson MA, O'Shea TM, Burda JE, Ao Y, Barlatey SL, Bernstein AM, Kim JH, James ND, Rogers A, Kato B, Wollenberg AL, Kawaguchi R, Coppola G, Wang C, Deming TJ, He Z, Courtine G, Sofroniew MV (2018) Required growth facilitators propel axon regeneration across complete spinal cord injury. Nature 561:396–400. 10.1038/s41586-018-0467-6 30158698 PMC6151128

[B3] Atwal JK, Pinkston-Gosse J, Syken J, Stawicki S, Wu Y, Shatz C, Tessier-Lavigne M (2008) PirB is a functional receptor for myelin inhibitors of axonal regeneration. Science 322:967–970. 10.1126/science.1161151 18988857

[B4] Avalos AM, Valdivia AD, Muñoz N, Herrera-Molina R, Tapia JC, Lavandero S, Chiong M, Burridge K, Schneider P, Quest AF, Leyton L (2009) Neuronal Thy-1 induces astrocyte adhesion by engaging syndecan-4 in a cooperative interaction with alphavbeta3 integrin that activates PKCalpha and RhoA. J Cell Sci 122:3462–3471. 10.1242/jcs.034827 19723805 PMC2746130

[B5] Bastiani MJ, Harrelson AL, Snow PM, Goodman CS (1987) Expression of fasciclin I and II glycoproteins on subsets of axon pathways during neuronal development in the grasshopper. Cell 48:745–755. 10.1016/0092-8674(87)90072-9 3545496

[B6] Bei F, Lee HHC, Liu X, Gunner G, Jin H, Ma L, Wang C, Hou L, Hensch TK, Frank E, Sanes JR, Chen C, Fagiolini M, He Z (2016) Restoration of visual function by enhancing conduction in regenerated axons. Cell 164:219–232. 10.1016/j.cell.2015.11.036 26771493 PMC4863988

[B7] Benowitz LI, Yin Y (2010) Optic nerve regeneration. Arch Ophthalmol 128:1059–1064. 10.1001/archophthalmol.2010.152 20697009 PMC3072887

[B8] Blackmore M, Letourneau PC (2006) L1, beta1 integrin, and cadherins mediate axonal regeneration in the embryonic spinal cord. J Neurobiol 66:1564–1583. 10.1002/neu.20311 17058193

[B9] Bray ER, Noga M, Thakor K, Wang Y, Lemmon VP, Park KK, Tsoulfas P (2017) 3D visualization of individual regenerating retinal ganglion cell axons reveals surprisingly complex growth paths. eNeuro 4:ENEURO.0093-17.2017 10.1523/ENEURO.0093-17.2017 PMC5575138 28856242

[B10] Bray ER, Yungher BJ, Levay K, Ribeiro M, Dvoryanchikov G, Ayupe AC, Thakor K, Marks V, Randolph M, Danzi MC, Schmidt TM, Chaudhari N, Lemmon VP, Hattar S, Park KK (2019) Thrombospondin-1 mediates axon regeneration in retinal ganglion cells. Neuron 103:642–657. 10.1016/j.neuron.2019.05.044 31255486 PMC6706310

[B11] Bruce FM, Brown S, Smith JN, Fuerst PG, Erskine L (2017) DSCAM promotes axon fasciculation and growth in the developing optic pathway. Proc Natl Acad Sci USA 114:1702–1707. 10.1073/pnas.1618606114 28137836 PMC5321013

[B12] Butt AM, Colquhoun K, Tutton M, Berry M (1994) Three-dimensional morphology of astrocytes and oligodendrocytes in the intact mouse optic nerve. J Neurocytol 23:469–485. 10.1007/BF01184071 7527074

[B13] Casper KB, Jones K, McCarthy KD (2007) Characterization of astrocyte-specific conditional knockouts. Genesis 45:292–299. 10.1002/dvg.20287 17457931

[B14] Chierzi S, Fawcett JW (2001) Regeneration in the mammalian optic nerve. Restor Neurol Neurosci 19:109–118. 12082232

[B15] Cho Y, Cavalli V (2012) HDAC5 is a novel injury-regulated tubulin deacetylase controlling axon regeneration. EMBO J 31:3063–3078. 10.1038/emboj.2012.160 22692128 PMC3400015

[B16] Chothia C, Jones EY (1997) The molecular structure of cell adhesion molecules. Annu Rev Biochem 66:823–862. 10.1146/annurev.biochem.66.1.823 9242926

[B17] Chung KH, Hart CC, Al-Bassam S, Avery A, Taylor J, Patel PD, Vojtek AB, Turner DL (2006) Polycistronic RNA polymerase II expression vectors for RNA interference based on BIC/miR-155. Nucleic Acids Res 34:e53. 10.1093/nar/gkl143 16614444 PMC1435982

[B18] de Lima S, Koriyama Y, Kurimoto T, Oliveira JT, Yin Y, Li Y, Gilbert HY, Fagiolini M, Martinez AM, Benowitz L (2012) Full-length axon regeneration in the adult mouse optic nerve and partial recovery of simple visual behaviors. Proc Natl Acad Sci USA 109:9149–9154. 10.1073/pnas.1119449109 22615390 PMC3384191

[B19] Domanskyi A, Geissler C, Vinnikov IA, Alter H, Schober A, Vogt MA, Gass P, Parlato R, Schütz G (2011) Pten ablation in adult dopaminergic neurons is neuroprotective in Parkinson’s disease models. FASEB J 25:2898–2910. 10.1096/fj.11-181958 21593433

[B20] Duan X, Qiao M, Bei F, Kim IJ, He Z, Sanes JR (2015) Subtype-specific regeneration of retinal ganglion cells following axotomy: effects of osteopontin and mTOR signaling. Neuron 85:1244–1256. 10.1016/j.neuron.2015.02.017 25754821 PMC4391013

[B21] Duffy P, Wang X, Siegel CS, Seigel CS, Tu N, Henkemeyer M, Cafferty WBJ, Strittmatter SM (2012) Myelin-derived ephrinB3 restricts axonal regeneration and recovery after adult CNS injury. Proc Natl Acad Sci USA 109:5063–5068. 10.1073/pnas.1113953109 22411787 PMC3323955

[B22] Eva R, Fawcett J (2014) Integrin signalling and traffic during axon growth and regeneration. Curr Opin Neurobiol 27:179–185. 10.1016/j.conb.2014.03.018 24793179

[B23] Fawcett JW (2017) An integrin approach to axon regeneration. Eye (Lond) 31:206–208. 10.1038/eye.2016.293 28009347 PMC5306470

[B24] Fawcett JW, Schwab ME, Montani L, Brazda N, Müller HW (2012) Defeating inhibition of regeneration by scar and myelin components. Handb Clin Neurol 109:503–522. 10.1016/B978-0-444-52137-8.00031-0 23098733

[B25] Ferguson TA, Scherer SS (2012) Neuronal cadherin (NCAD) increases sensory neurite formation and outgrowth on astrocytes. Neurosci Lett 522:108–112. 10.1016/j.neulet.2012.06.013 22698587 PMC3784833

[B26] Fischer D (2017) Hyper-IL-6: a potent and efficacious stimulator of RGC regeneration. Eye (Lond) 31:173–178. 10.1038/eye.2016.234 27886185 PMC5306455

[B27] Geoffroy CG, Zheng B (2014) Myelin-associated inhibitors in axonal growth after CNS injury. Curr Opin Neurobiol 27:31–38. 10.1016/j.conb.2014.02.012 24608164 PMC4122599

[B28] Graham HK, Duan X (2020) Molecular mechanisms regulating synaptic specificity and retinal circuit formation. Wiley Interdiscip Rev Dev Biol 8:e379. 10.1002/wdev.379 PMC7541429 32267095

[B29] Hansen SM, Berezin V, Bock E (2008) Signaling mechanisms of neurite outgrowth induced by the cell adhesion molecules NCAM and N-cadherin. Cell Mol Life Sci 65:3809–3821. 10.1007/s00018-008-8290-0 18791849 PMC11131707

[B30] Hara M, Kobayakawa K, Ohkawa Y, Kumamaru H, Yokota K, Saito T, Kijima K, Yoshizaki S, Harimaya K, Nakashima Y, Okada S (2017) Interaction of reactive astrocytes with type I collagen induces astrocytic scar formation through the integrin-N-cadherin pathway after spinal cord injury. Nat Med 23:818–828. 10.1038/nm.4354 28628111

[B31] Hillen AEJ, Burbach JPH, Hol EM (2018) Cell adhesion and matricellular support by astrocytes of the tripartite synapse. Prog Neurobiol 165-167:66–86. 10.1016/j.pneurobio.2018.02.002 29444459

[B32] Izumi Y, Wakita S, Kanbara C, Nakai T, Akaike A, Kume T (2017) Integrin α5β1 expression on dopaminergic neurons is involved in dopaminergic neurite outgrowth on striatal neurons. Sci Rep 7:42111. 10.1038/srep42111 28176845 PMC5296761

[B33] Kamiguchi H (2007) The role of cell adhesion molecules in axon growth and guidance. Adv Exp Med Biol 621:95–103. 10.1007/978-0-387-76715-4_7 18269213

[B34] Leaver SG, Cui Q, Plant GW, Arulpragasam A, Hisheh S, Verhaagen J, Harvey AR (2006) AAV-mediated expression of CNTF promotes long-term survival and regeneration of adult rat retinal ganglion cells. Gene Ther 13:1328–1341. 10.1038/sj.gt.3302791 16708079

[B35] Leibinger M, Andreadaki A, Diekmann H, Fischer D (2013) Neuronal STAT3 activation is essential for CNTF- and inflammatory stimulation-induced CNS axon regeneration. Cell Death Dis 4:e805. 10.1038/cddis.2013.310 24052073 PMC3789169

[B36] Leibinger M, Hilla AM, Andreadaki A, Fischer D (2019) GSK3-CRMP2 signaling mediates axonal regeneration induced by Pten knockout. Commun Biol 2:318. 10.1038/s42003-019-0524-1 31453382 PMC6707209

[B37] Lelièvre EC, Plestant C, Boscher C, Wolff E, Mege RM, Birbes H (2012) N-cadherin mediates neuronal cell survival through Bim down-regulation. PLoS One 7:e33206. 10.1371/journal.pone.0033206 22427990 PMC3299760

[B38] Lim JH, Stafford BK, Nguyen PL, Lien BV, Wang C, Zukor K, He Z, Huberman AD (2016) Neural activity promotes long-distance, target-specific regeneration of adult retinal axons. Nat Neurosci 19:1073–1084. 10.1038/nn.4340 27399843 PMC5708130

[B39] Liu K, Lu Y, Lee JK, Samara R, Willenberg R, Sears-Kraxberger I, Tedeschi A, Park KK, Jin D, Cai B, Xu B, Connolly L, Steward O, Zheng B, He Z (2010) PTEN deletion enhances the regenerative ability of adult corticospinal neurons. Nat Neurosci 13:1075–1081. 10.1038/nn.2603 20694004 PMC2928871

[B40] Luo X, Salgueiro Y, Beckerman SR, Lemmon VP, Tsoulfas P, Park KK (2013) Three dimensional evaluation of retinal ganglion cell axon regeneration and pathfinding in whole mouse tissue after injury. Exp Neurol 247:653–662. 10.1016/j.expneurol.2013.03.001 23510761 PMC3726550

[B41] Luo X, Ribeiro M, Bray ER, Lee DH, Yungher BJ, Mehta ST, Thakor KA, Diaz F, Lee JK, Moraes CT, Bixby JL, Lemmon VP, Park KK (2016) Enhanced transcriptional activity and mitochondrial localization of STAT3 co-induce axon regrowth in the adult central nervous system. Cell Rep 15:398–410. 10.1016/j.celrep.2016.03.029 27050520 PMC4833545

[B42] Maître JL, Heisenberg CP (2013) Three functions of cadherins in cell adhesion. Curr Biol 23:R626–R633. 10.1016/j.cub.2013.06.019 23885883 PMC3722483

[B43] Matsunaga M, Hatta K, Nagafuchi A, Takeichi M (1988) Guidance of optic nerve fibres by N-cadherin adhesion molecules. Nature 334:62–64. 10.1038/334062a0 3386742

[B44] McKerracher L, Rosen KM (2015) MAG, myelin and overcoming growth inhibition in the CNS. Front Mol Neurosci 8:51. 10.3389/fnmol.2015.00051 26441514 PMC4561339

[B45] Meager A (1999) Cytokine regulation of cellular adhesion molecule expression in inflammation. Cytokine Growth Factor Rev 10:27–39. 10.1016/s1359-6101(98)00024-0 10379910

[B46] Missaire M, Hindges R (2015) The role of cell adhesion molecules in visual circuit formation: from neurite outgrowth to maps and synaptic specificity. Dev Neurobiol 75:569–583. 10.1002/dneu.22267 25649254 PMC4855686

[B47] Moore DL, Goldberg JL (2011) Multiple transcription factor families regulate axon growth and regeneration. Dev Neurobiol 71:1186–1211. 10.1002/dneu.20934 21674813 PMC3212623

[B48] Moore DL, Blackmore MG, Hu Y, Kaestner KH, Bixby JL, Lemmon VP, Goldberg JL (2009) KLF family members regulate intrinsic axon regeneration ability. Science 326:298–301. 10.1126/science.1175737 19815778 PMC2882032

[B49] Müller A, Hauk TG, Leibinger M, Marienfeld R, Fischer D (2009) Exogenous CNTF stimulates axon regeneration of retinal ganglion cells partially via endogenous CNTF. Mol Cell Neurosci 41:233–246. 10.1016/j.mcn.2009.03.002 19332123

[B50] Nathan FM, Ohtake Y, Wang S, Jiang X, Sami A, Guo H, Zhou FQ, Li S (2020) Upregulating Lin28a promotes axon regeneration in adult mice with optic nerve and spinal cord injury. Mol Ther 28:1902–1917. 32353321 10.1016/j.ymthe.2020.04.010 PMC7403348

[B51] Nguyen PT, Nguyen D, Chea C, Miyauchi M, Fujii M, Takata T (2018) Interaction between N cadherin and decoy receptor-2 regulates apoptosis in head and neck cancer. Oncotarget 9:31516–31530. 10.18632/oncotarget.25846 30140387 PMC6101147

[B52] Nieuwenhuis B, Haenzi B, Andrews MR, Verhaagen J, Fawcett JW (2018) Integrins promote axonal regeneration after injury of the nervous system. Biol Rev Camb Philos Soc 93:1339–1362. 10.1111/brv.12398 29446228 PMC6055631

[B53] Park K, Luo JM, Hisheh S, Harvey AR, Cui Q (2004) Cellular mechanisms associated with spontaneous and ciliary neurotrophic factor-cAMP-induced survival and axonal regeneration of adult retinal ganglion cells. J Neurosci 24:10806–10815. 10.1523/JNEUROSCI.3532-04.2004 15574731 PMC6730205

[B54] Park KK, Liu K, Hu Y, Smith PD, Wang C, Cai B, Xu B, Connolly L, Kramvis I, Sahin M, He Z (2008) Promoting axon regeneration in the adult CNS by modulation of the PTEN/mTOR pathway. Science 322:963–966. 10.1126/science.1161566 18988856 PMC2652400

[B55] Park YM, Chun H, Shin JI, Lee CJ (2018) Astrocyte specificity and coverage of hGFAP-CreERT2 [Tg(GFAP-Cre/ERT2)13Kdmc] mouse line in various brain regions. Exp Neurobiol 27:508–525. 10.5607/en.2018.27.6.508 30636902 PMC6318562

[B56] Pernet V, Schwab ME (2012) The role of Nogo-A in axonal plasticity, regrowth and repair. Cell Tissue Res 349:97–104. 10.1007/s00441-012-1432-6 22588543

[B57] Pernet V, Schwab ME (2014) Lost in the jungle: new hurdles for optic nerve axon regeneration. Trends Neurosci 37:381–387. 10.1016/j.tins.2014.05.002 24874558

[B58] Pernet V, Joly S, Jordi N, Dalkara D, Guzik-Kornacka A, Flannery JG, Schwab ME (2013) Misguidance and modulation of axonal regeneration by Stat3 and Rho/ROCK signaling in the transparent optic nerve. Cell Death Dis 4:e734. 10.1038/cddis.2013.266 23868067 PMC3730436

[B59] Radice GL, Rayburn H, Matsunami H, Knudsen KA, Takeichi M, Hynes RO (1997) Developmental defects in mouse embryos lacking N-cadherin. Dev Biol 181:64–78. 10.1006/dbio.1996.8443 9015265

[B60] Redies C (2000) Cadherins in the central nervous system. Prog Neurobiol 61:611–648. 10.1016/s0301-0082(99)00070-2 10775799

[B61] Redies C, Takeichi M (1996) Cadherins in the developing central nervous system: an adhesive code for segmental and functional subdivisions. Dev Biol 180:413–423. 10.1006/dbio.1996.0315 8954714

[B62] Rheaume BA, Jereen A, Bolisetty M, Sajid MS, Yang Y, Renna K, Sun L, Robson P, Trakhtenberg EF (2018) Single cell transcriptome profiling of retinal ganglion cells identifies cellular subtypes. Nat Commun 9:2759. 10.1038/s41467-018-05134-3 30018341 PMC6050223

[B63] Rigby MJ, Gomez TM, Puglielli L (2020) Glial cell-axonal growth cone interactions in neurodevelopment and regeneration. Front Neurosci 14:203. 10.3389/fnins.2020.00203 32210757 PMC7076157

[B64] Rodriguez AR, de Sevilla Müller LP, Brecha NC (2014) The RNA binding protein RBPMS is a selective marker of ganglion cells in the mammalian retina. J Comp Neurol 522:1411–1443. 10.1002/cne.23521 24318667 PMC3959221

[B65] Schwabe T, Borycz JA, Meinertzhagen IA, Clandinin TR (2014) Differential adhesion determines the organization of synaptic fascicles in the *Drosophila* visual system. Curr Biol 24:1304–1313. 10.1016/j.cub.2014.04.047 24881879 PMC4500537

[B66] Shapiro L, Fannon AM, Kwong PD, Thompson A, Lehmann MS, Grübel G, Legrand JF, Als Nielsen J, Colman DR, Hendrickson WA (1995) Structural basis of cell-cell adhesion by cadherins. Nature 374:327–337. 10.1038/374327a0 7885471

[B67] Sharma K, Selzer ME, Li S (2012) Scar-mediated inhibition and CSPG receptors in the CNS. Exp Neurol 237:370–378. 10.1016/j.expneurol.2012.07.009 22836147 PMC5454774

[B68] Silver J, Schwab ME, Popovich PG (2014) Central nervous system regenerative failure: role of oligodendrocytes, astrocytes, and microglia. Cold Spring Harb Perspect Biol 7:a020602. 10.1101/cshperspect.a020602 25475091 PMC4355267

[B69] Sun F, Park KK, Belin S, Wang D, Lu T, Chen G, Zhang K, Yeung C, Feng G, Yankner BA, He Z (2011) Sustained axon regeneration induced by co-deletion of PTEN and SOCS3. Nature 480:372–375. 10.1038/nature10594 22056987 PMC3240702

[B70] Tan CL, Kwok JC, Patani R, Ffrench-Constant C, Chandran S, Fawcett JW (2011) Integrin activation promotes axon growth on inhibitory chondroitin sulfate proteoglycans by enhancing integrin signaling. J Neurosci 31:6289–6295. 10.1523/JNEUROSCI.0008-11.2011 21525268 PMC3116378

[B71] Tedeschi A, Dupraz S, Laskowski CJ, Xue J, Ulas T, Beyer M, Schultze JL, Bradke F (2016) The calcium channel subunit alpha2delta2 suppresses axon regeneration in the adult CNS. Neuron 92:419–434. 10.1016/j.neuron.2016.09.026 27720483

[B72] Tom VJ, Doller CM, Malouf AT, Silver J (2004) Astrocyte-associated fibronectin is critical for axonal regeneration in adult white matter. J Neurosci 24:9282–9290. 10.1523/JNEUROSCI.2120-04.2004 15496664 PMC6730112

[B73] Tomaselli KJ, Neugebauer KM, Bixby JL, Lilien J, Reichardt LF (1988) N-cadherin and integrins: two receptor systems that mediate neuronal process outgrowth on astrocyte surfaces. Neuron 1:33–43. 10.1016/0896-6273(88)90207-3 2856086

[B74] Tran NL, Adams DG, Vaillancourt RR, Heimark RL (2002) Signal transduction from N-cadherin increases Bcl-2. Regulation of the phosphatidylinositol 3-kinase/Akt pathway by homophilic adhesion and actin cytoskeletal organization. J Biol Chem 277:32905–32914. 10.1074/jbc.M200300200 12095980

[B75] Tran NM, Shekhar K, Whitney IE, Jacobi A, Benhar I, Hong G, Yan W, Adiconis X, Arnold ME, Lee JM, Levin JZ, Lin D, Wang C, Lieber CM, Regev A, He Z, Sanes JR (2019) Single-cell profiles of retinal ganglion cells differing in resilience to injury reveal neuroprotective genes. Neuron 104:1039–1055. 10.1016/j.neuron.2019.11.006 31784286 PMC6923571

[B76] Vendome J, Posy S, Jin X, Bahna F, Ahlsen G, Shapiro L, Honig B (2011) Molecular design principles underlying β-strand swapping in the adhesive dimerization of cadherins. Nat Struct Mol Biol 18:693–700. 10.1038/nsmb.2051 21572446 PMC3113550

[B77] Walsh FS, Doherty P (1997) Neural cell adhesion molecules of the immunoglobulin superfamily: role in axon growth and guidance. Annu Rev Cell Dev Biol 13:425–456. 10.1146/annurev.cellbio.13.1.425 9442880

[B78] Wang XW, Li Q, Liu CM, Hall PA, Jiang JJ, Katchis CD, Kang S, Dong BC, Li S, Zhou FQ (2018) Lin28 signaling supports mammalian PNS and CNS axon regeneration. Cell Rep 24:2540–2552. 10.1016/j.celrep.2018.07.105 30184489 PMC6173831

[B79] Wang XW, Yang SG, Zhang C, Hu MW, Qian J, Ma JJ, Zhang Y, Yang BB, Weng YL, Ming GL, Kosanam AR, Saijilafu, Zhou FQ (2020) Knocking out non-muscle myosin II in retinal ganglion cells promotes long-distance optic nerve regeneration. Cell Rep 31:107537. 10.1016/j.celrep.2020.107537 32320663 PMC7219759

[B80] Wiese S, Karus M, Faissner A (2012) Astrocytes as a source for extracellular matrix molecules and cytokines. Front Pharmacol 3:120. 10.3389/fphar.2012.00120 22740833 PMC3382726

[B81] Yang L, Miao L, Liang F, Huang H, Teng X, Li S, Nuriddinov J, Selzer ME, Hu Y (2014) The mTORC1 effectors S6K1 and 4E-BP play different roles in CNS axon regeneration. Nat Commun 5:5416. 10.1038/ncomms6416 25382660 PMC4228696

[B82] Yang SG, Li CP, Peng XQ, Teng ZQ, Liu CM, Zhou FQ (2020) Strategies to promote long distance optic nerve regeneration. Front Cell Neurosci 14:119. 10.3389/fncel.2020.00119 32477071 PMC7240020

[B83] Yin Y, Henzl MT, Lorber B, Nakazawa T, Thomas TT, Jiang F, Langer R, Benowitz LI (2006) Oncomodulin is a macrophage-derived signal for axon regeneration in retinal ganglion cells. Nat Neurosci 9:843–852. 10.1038/nn1701 16699509

[B84] Yonekura S, Ting CY, Neves G, Hung K, Hsu SN, Chiba A, Chess A, Lee CH (2006) The variable transmembrane domain of *Drosophila* N-cadherin regulates adhesive activity. Mol Cell Biol 26:6598–6608. 10.1128/MCB.00241-06 16914742 PMC1592838

[B85] Yungher BJ, Luo X, Salgueiro Y, Blackmore MG, Park KK (2015) Viral vector-based improvement of optic nerve regeneration: characterization of individual axons’ growth patterns and synaptogenesis in a visual target. Gene Ther 22:811–821. 10.1038/gt.2015.51 26005861 PMC4600032

[B86] Zukor K, Belin S, Wang C, Keelan N, Wang X, He Z (2013) Short hairpin RNA against PTEN enhances regenerative growth of corticospinal tract axons after spinal cord injury. J Neurosci 33:15350–15361. 10.1523/JNEUROSCI.2510-13.2013 24068802 PMC3782617

